# Molecular Hybrid Design, Synthesis, In Vitro Cytotoxicity, In Silico ADME and Molecular Docking Studies of New Benzoate Ester-Linked Arylsulfonyl Hydrazones

**DOI:** 10.3390/molecules29153478

**Published:** 2024-07-25

**Authors:** Erdem Ergan, Reşit Çakmak, Eyüp Başaran, Suraj N. Mali, Senem Akkoc, Sivakumar Annadurai

**Affiliations:** 1 Department of Property Protection and Security, Van Security Vocational School, Van Yuzuncu Yil University, Van 65080, Türkiye; 2 Medical Laboratory Techniques Program, Vocational School of Health Services, Batman University, Batman 72060, Türkiye; resit.cakmak@batman.edu.tr; 3 Department of Chemistry and Chemical Processing Technologies, Vocational School of Technical Sciences, Batman University, Batman 72060, Türkiye; 4 School of Pharmacy, D.Y. Patil University (Deemed to be University), Sector 7, Nerul, Navi Mumbai 400706, India; mali.suraj1695@gmail.com; 5 Department of Basic Pharmaceutical Sciences, Faculty of Pharmacy, Suleyman Demirel University, Isparta 32260, Türkiye; senemakkoc@sdu.edu.tr; 6 Faculty of Engineering and Natural Sciences, Bahcesehir University, Istanbul 34353, Türkiye; 7 Department of Pharmacognosy, College of Pharmacy, King Khalid University, Abha 61421, Saudi Arabia; sannadurai@kku.edu.sa

**Keywords:** sulfonyl hydrazone, chemotherapeutic agent, antiproliferative activity, in silico study

## Abstract

In this paper, we present the synthesis and characterization of two known sulfonyl hydrazides (**1** and **2**) and their new sulfonyl hydrazone derivatives (**9**–**20**), as well as in vitro and in silico investigations of their cytotoxic properties against human lung (A549) and human breast (MCF-7) cancer cell lines. The target compounds (**9**–**20**) obtained in high yields were synthesized for the first time by a multi-step reaction, and their structures were confirmed by elemental analysis and various spectral techniques, including FT-IR, ^1^H-, and ^13^C-NMR. The antiproliferative profiles of these compounds (**1**, **2**, and **9**–**20**) in this study were determined at concentrations of 200, 100, 50, and 25 µM against selected cancer cell lines for 72 h using the 3-(4,5-dimethylthiazol-2-yl)-2,5-diphenyl tetrazolium bromide (MTT) method. Except for compounds **1** and **2**, other compounds (**9**–**20**) demonstrated cytotoxic activity at concentrations lower than 200 µM. The newly synthesized compounds (**9**–**20**) demonstrated antiproliferative activities at a micromolar level, with IC_50_ values in the range of 29.59–176.70 μM for the A549 cell line and 27.70–170.30 μM for the MCF-7 cell line. Among these compounds, compound **15** (IC_50_ = 29.59 μM against A549 cell line and IC_50_ = 27.70 μM against MCF-7 cell line) showed the highest cytotoxic activity against these two cancer cell lines compared to the reference drug cisplatin (IC_50_ = 22.42 μM against A549 cell line and IC_50_ = 18.01 μM against MCF-7 cell line). From docking simulations, to establish a plausible binding mode of compounds, we noticed that compound **15** demonstrated the highest affinity (−6.8508 kcal/mol) for estrogen receptor-beta (ERbeta) compared to others, suggesting promising ERbeta binding potential. Most compounds followed Lipinski’s rule of five, with acceptable logP values. Additionally, all had mixed gastrointestinal absorption and limited blood–brain barrier permeability. Overall, our study proposed new sulfonyl hydrazones as a potential class of anticancer agents.

## 1. Introduction

Cancer manifests because of the unregulated division and proliferation of cells within an organ or tissue, presenting itself as a significant public health and economic challenge in the contemporary era. Currently, the increase in the main risk factors that trigger cancer (such as smoking, being overweight, obesity, and infection) and the increase in average life expectancy have led to an increase in cancer-related deaths. According to data from the International Agency for Research on Cancer (IARC), approximately 20 million new cancer cases were diagnosed worldwide in 2022, and 9.7 million deaths occurred due to cancer. This disease is responsible for approximately one in six deaths and one in four deaths from noncommunicable diseases worldwide. According to IARC data, it is estimated that the number of new cancer cases will reach 35 million by 2050 [1,2]. 

In humans, lung and breast cancers exhibit the highest incidence and mortality rates among women and men, respectively, compared to other forms of cancer. Lung cancer was the most commonly diagnosed cancer in 2022, responsible for almost 2.5 million new cases or one in eight cancers worldwide. This was followed by cancers of the breast (11.6%) and colorectal cancer (9.6%). Lung cancer has emerged as the primary contributor to cancer mortality, accounting for approximately 1.8 million fatalities (18.7%). Subsequently, colorectal (9.3%), liver (7.8%), breast (6.9%), and stomach (6.8%) cancers followed suit [1,2,3,4].

Current cancer treatment approaches include methods such as chemotherapy, surgery, radiotherapy, hormone therapy, and immunotherapy. One or more of these methods is used in clinical practice for patients with cancer. Despite its efficacy as a therapeutic modality, chemotherapy is widely recognized as a treatment associated with numerous adverse effects owing to its deleterious impact on non-cancerous cells. Consequently, in light of the increasing prevalence of cancer, extensive efforts are currently underway to explore novel anticancer compounds capable of inducing apoptosis in malignant cells and inhibiting their replicative capacity [5,6,7,8,9].

**Figure 1:**
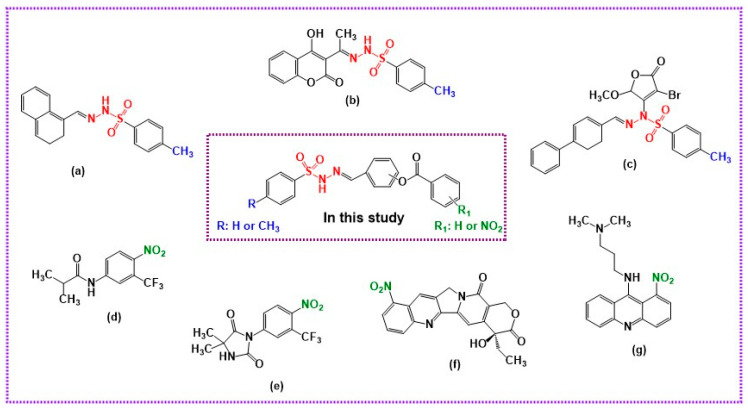
Graphical representation of the design strategy of the targeted compounds.

Hydrazones are a remarkable class of biologically active compounds that have attracted the attention of medicinal chemists owing to their many pharmacological properties. In recent years, many researchers have been designing and synthesizing these molecules to obtain drugs with the least toxicity and the greatest possible therapeutic effect for use in the treatment of some important diseases [10]. In the literature, researchers have reported in some studies that compounds carrying the hydrazide–hydrazone moiety have different pharmacological activities [11,12,13]. Among these, iproniazid and isocarboxazid are commercial products obtained by reducing this chemical group. In addition, nifuroxazid is an example of a commercial product that also carries this pharmacophore group [14]. Iproniazid is used in the treatment of tuberculosis [15]. Its antidepressant effects have also been proven. Nifuroxazide is another hydrazide–hydrazone with proven therapeutic efficacy when used as an intestinal antiseptic [16,17]. Regarding these compounds, it has been reported that the protons (-NHN=CH-) in the azomethine part of their structure cause therapeutic effects [18]. On the other hand, sulfonyl hydrazones have attracted great attention in recent years due to their numerous potential uses in medicinal chemistry [19]. The pharmacological properties of these compounds, such as enzymatic modulation [20], analgesia [21], antifungal aspects [22], and antibacterial potential [23,24], are essentially well known. In addition, these compounds have a wide range of bioactivities, including anticancer [25,26,27,28,29,30], antidepressant [31,32], and anticholinesterase activities against Alzheimer’s disease [33,34]. Among the sulfonyl hydrazone derivatives with anticancer activity, 4-methylbenzene sulfonyl hydrazone derivatives (compounds **a** [25], **b** [28], and **c** [27] (Figure 1) were determined to show varying ranges of cytotoxic activity against various cancer cell lines.

Nitro compounds, which are important intermediates in synthetic organic chemistry, are among the classes of compounds that have important biological and pharmacological effects. To date, a significant number of these compounds have shown various biological activities, including anticancer activity, and some of these compounds are still used as drugs in the treatment of many diseases, including cancer [35,36,37,38,39,40,41]. Examples of drugs containing nitro groups used in cancer treatment include flutamide (**d**) and nilutamide (**e**) (Figure 1). These drugs, which carry a nitro group in the benzene ring, are widely used for the treatment of prostate cancer [36,39]. Rubitecan (**f**) and nitracrine (**g**) are anticancer drugs that contain nitro groups (Figure 1). Rubitecan is a derivative of camptothecin. Nitracrine is an acridine derivative. These drugs are used to treat various cancers [36]. Inspired by the information given above, to discover new and potent anticancer drug candidates, we aimed to synthesize new anticancer drug candidate compounds bearing sulfonyl hydrazone and nitroaromatic moieties as pharmacophores and to evaluate their cytotoxic potential against A549 and MCF-7 cancer cell lines. Herein, the newly synthesized compounds were characterized spectroscopically using spectroscopic techniques (^1^H NMR, ^13^C NMR, and FT-IR) and elemental analysis. Our drug design strategy for the molecules targeted in this study was based on investigating the effects of methyl and nitro groups in the molecular skeleton on the antiproliferative activities of newly synthesized hybrid molecules against these cancer cell lines, as shown in Figure 1. 

Over the past several decades, computer-aided drug design (CADD) applications have been found to play a role in establishing the theoretical binding modes of lead molecules. We also screened these compounds for their in silico pharmacokinetics and drug-likeness characteristics using a well-known web server.

## 2. Results and Discussion

### 2.1. Synthesis and Characterization

**Scheme 1:**
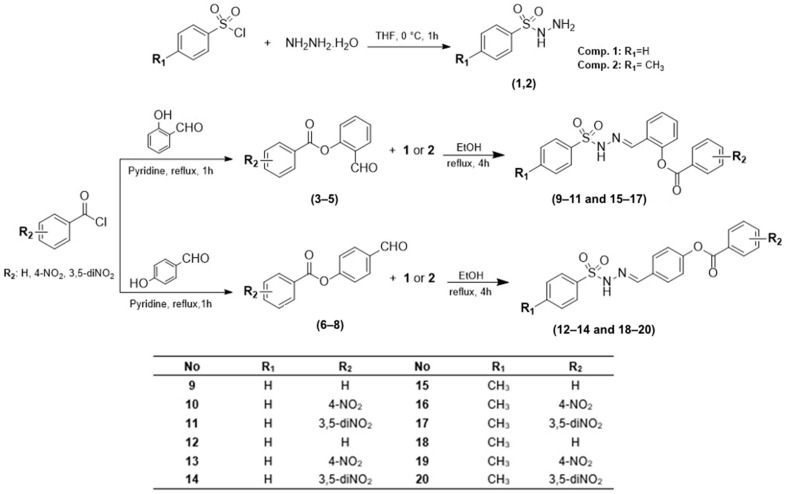
General synthesis pathway for new sulfonyl hydrazones (**9**–**20**).

In this study, to explore new anticancer agents, the targeted compounds, sulfonyl hydrazones, (**9**–**20**) were synthesized in good yields in three steps. The synthetic strategy for these molecules is summarized in Scheme 1. As shown in Scheme 1, the target compounds were synthesized by reacting aryl esters (**3**–**8**) with benzenesulfonyl hydrazide (**1**) and *p*-toluenesulfonyl hydrazide (**2**). In the first step, compounds **1** and **2** were successfully synthesized by treating of benzenesulfonyl chloride and *p*-toluenesulfonyl chloride with hydrazine hydrate in tetrahydrofuran (THF) as a solvent. In the second step, aryl ester compounds (**3**–**8**) were obtained by reacting three unsubstituted and nitro-substituted benzoyl chloride derivatives with 2-hydroxybenzaldehyde and 4-hydroxybenzaldehyde, respectively, in dichloromethane in the presence of triethylamine. In the final step, target molecules (**9**–**20**) were obtained by reacting aryl esters with compounds **1** and **2** in an ethanolic solution. Among the synthesized molecules, hydrazide derivatives (**1** and **2**) were known. Benzoate derivatives (**3**–**8**) were synthesized and characterized in our previous study [34,35]. On the other hand, hydrazone compounds (**9**–**20**) were synthesized for the first time as target molecules.

In the present study, the targeted molecules (**1**, **2**, and **9**–**20**) were characterized by elemental analysis and various spectroscopic methods, including FT-IR, and ^1^H- and ^13^C NMR. In the FT-IR spectra of the newly synthesized sulfonyl hydrazones (**9**–**20**), the NH stretching bands were in the range of 3271–3172 cm^−1^. The carbonyl group (C=O) stretching bands of the benzoate moiety of the compounds showed sharp vibrations in the range of 1744–1706 cm^−1^. The stretching bands of the imine group (C=N), indicating the presence of azomethine, the most prominent functional group in the compounds, were also observed in the range of 1629–1595 cm^−1^. Asymmetric and symmetric stretching vibration bands of the SO_2_ group were detected in the FT-IR spectra in the ranges of 1342–1319 cm^−1^ and 1174–1158 cm^−1^, respectively. In addition, asymmetric and symmetric stretching vibration bands of the NO_2_ groups in the structures of compounds (**10**, **11**, **13**, **14**, **16**, **17**, **19**, and **20**) were detected in the ranges of 1547–1514 cm^−1^ and 1370–1327 cm^−1^, respectively. In the ^1^H NMR spectra of the obtained target products, singlet SO_2_NH protons were observed to be resonant in the range of δ 11.47 to 11.61 ppm. The azomethine protons (CH=N) resonated in the range of δ 7.94–8.02 ppm indicating the formation of the hydrazone structure, is clear evidence that the target compounds have been synthesized. Moreover, the protons of aromatic phenyl rings were found to resonate between δ 7.28 ppm and δ 9.14 ppm. The methyl (–CH_3_) protons of compounds **15**–**20** were observed to resonate as singlets in the range of δ 2.31–2.35 ppm. In the ^13^C NMR spectra, the C=O carbon resonated in the range of δ 161.79–164.89 ppm while the C=N carbon resonated in the range of δ 148.11–152.29 ppm. Aromatic carbons resonated in the expected field. Thus, the characterization and elemental analysis data of these compounds were compatible with the proposed molecular structures (See Supplementary Materials). 

### 2.2. The Cytotoxic Activity Studies

Currently, chemotherapy is the most commonly used approach for the treatment of cancer. Available drugs have many side effects, such as low selectivity, toxicity, and acquired drug resistance [42,43]. Therefore, there is an urgent need to develop new and potent anticancer agents. Lung and breast cancers, which have different biological characteristics and treatment approaches, are among the most common cancer types worldwide and cause the most deaths [44]. Therefore, in this study, the in vitro cytotoxic activities of all the synthesized compounds (**1**, **2**, and **9**–**20**) were investigated against A549 and MCF-7 cell lines. The cells were treated with different concentrations of the molecules (200, 100, 50, and 25 µM) for 72 h and the IC_50_ values were determined. The results are presented in Table 1.

The IC_50_ values indicate the concentration of the molecule required to inhibit cell viability by 50%. Lower IC_50_ values denote higher cytotoxicity. 

Table 1 shows that, except for one (**1**) of the molecules synthesized, thirteen others (**2** and **9**–**20**) had cytotoxic effects against the A549 cell line. Modifications in the molecular structure of molecules (**1**, **2**, and **9**–**20**) can significantly influence their cytotoxic properties. For instance, molecule **10**, having a 4-nitrobenzoyl group, with IC_50_ values of 58.83 µM and 34.61 µM against A549 and MCF-7 cell lines, respectively, appears to be more effective than other derivatives such as molecules **13** and **14**. These results demonstrate the significance of the substituents on the core of the molecule. Thus, the structural attributes contribute to the cytotoxic effects of these molecules. Molecule **9**, having a benzoyl group, and **17**, having a 3,5-dinitrobenzoyl group, showed very similar cytotoxic effect values against the A549 cell line with IC_50_ values of 50.72 and 50.91 µM, respectively. When compounds **18**–**20** were compared among themselves, it was found that both compounds containing nitro groups (**19**, **20**) had lower effects in A549 than compound **18**, with IC_50_ values of 166.20 and 128.30 µM, respectively. A similar situation was observed for compounds **9**–**11**, **12**–**14**, and **15**–**17**. 

The results of the cytotoxic activity studies revealed significant variations in the efficacy of the molecules against A549 and MCF-7 cell lines. According to the structure activity relationship, it can be seen in Table 1 that the molecules showing the lowest cytotoxic effect are among the series containing the 4-methylbenzenesulfonhydrazide group. Several compounds, particularly molecule **15,** containing benzoyl and 4-methylbenzenesulfonhydrazide groups demonstrated potent cytotoxic effects, with IC_50_ values of 29.59 µM and 27.70 µM being found in the A549 and MCF-7 cell lines, respectively. These values are noteworthy when compared to the standard chemotherapeutic agent, cisplatin, which exhibited an IC_50_ of 22.42 µM against A549 cells. The greater effectiveness of **15** against both A549 and MCF-7 cells may be due to enhanced cellular uptake or interaction with critical cellular targets involved in cell proliferation and survival. In particular, the promising cytotoxic profile of **15** underscores its potential as a lead compound for further development in cancer therapy.

**Figure 2:**
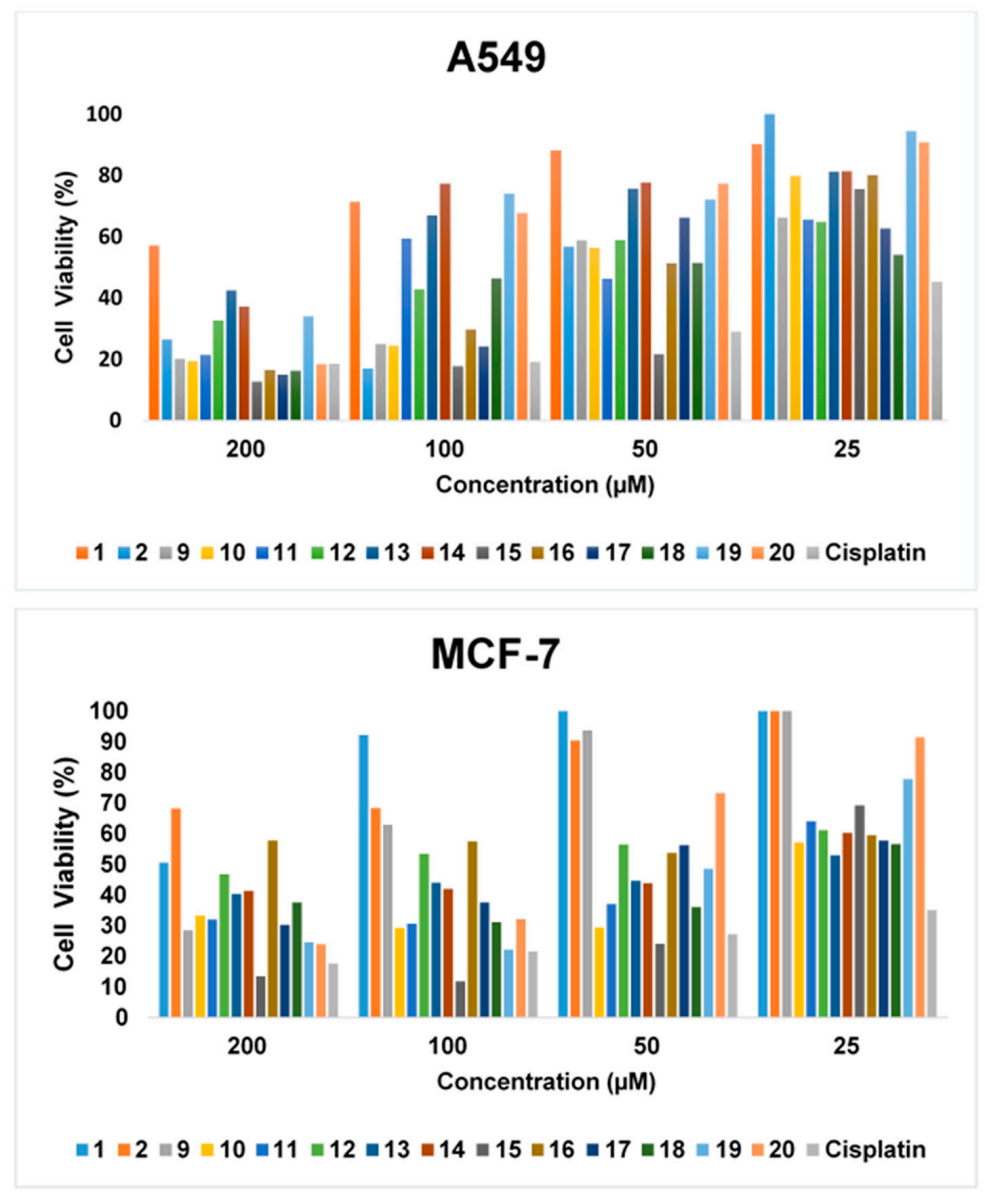
Cell viability ratio depending on concentrations of **1**, **2**, and **9**–**20**.

Benzenesulfonyl chloride and 4-methylbenzenesulfonyl chloride used as starting materials did not show cytotoxic effects against the MCF-7 cell line at the studied concentrations and incubation periods. All the new molecules (**9**–**20**) synthesized using these (**1**, **2**) were found to be effective in inhibiting the growth of cells in the breast cancer cell line. Compounds **10**, **11**, **13**, **14**, **15**, **18**, **19**, and **20** showed higher effects in the MCF-7 cell line than in the A549 cell line, with IC_50_ values of 34.61, 64.96, 54.30, 59.92, 27.70, 40.58, 51.03, and 86.06 µM, respectively. The varying viability rates of A549 and MCF-7 cells, depending on the concentrations of the drug candidates, are shown in Figure 2.

At a 200 μM concentration of drug candidates, the viability rates of A549 cells were obtained as 57.22%, 26.51%, 20.20%, 19.43%, 21.51%, 32.69%, 42.49%, 37.24%, 12.72%, 16.60%, 14.98%, 16.27%, 34.17%, and 18.37% for compounds **1**, **2**, and **9**–**20)**, respectively. These cell viability rates increased further at 25 μM and were determined as 90.17%, 100%, 66.24%, 79.91%, 65.62%, 64.92%, 81.28%, 81.38%, 75.56%, 80.25%, 62.68%, 54.22%, 94.52%, and 90.85% for compounds **1**, **2**, and **9**–**20**, respectively. 

### 2.3. Molecular Docking Studies

Recent global studies have focused on developing new cancer drugs due to rising patient numbers, drug side effects, toxicity, and tumor resistance [45]. Chemotherapy, a common treatment, targets tumors but often causes severe side effects. There is a pressing need for safer anticancer agents that can halt or eliminate cancer cells without harming healthy ones [45]. Breast cancer, the second most prevalent cancer worldwide and the most common among women, encompasses various subtypes with unique gene expression and survival rates [45]. Advances in treatment stem from biomarker discoveries, facilitating tailored therapies for different molecular subgroups. Estrogen-dependent breast cancer cells, such as MCF7 and T47D cells, rely on estrogen for growth. Anti-estrogen treatment halts proliferation and induces apoptosis. Estrogen is vital for mammary gland development and promotes the growth of breast cancer. The effects of estrogen are mediated by two receptors, estrogen receptors (ERα) and ERβ. ERβ1, discovered later, exhibits antiproliferative effects when introduced into ERα-positive breast cancer cells, as observed in T47D cells expressing ERβ1 [45]. Furthermore, from the previous literature, it was noticed that hydrazones had stronger inhibitory potentials against MCF-7 cell lines [46]. The choice of pdb id selection also relied on a previous literature source [46]. Molecular docking simulation studies explored the potential binding mechanisms or modes for the synthesized molecules. Based on the aforesaid background, we found it worthwhile to investigate the docking potentials of synthesized Sulfonyl hydrazones against the chosen target, estrogen receptor-beta (ERβ) (pdb id: 1x7b; Resolution: 2.30 Å; R-Value Work: 0.218). For a better comparison, we used doxorubicin, an often-used anticancer medicine. 

From docking simulations, it was observed that molecule (**1**) retained the lowest docking affinity towards the selected target (docking score: −4.3304 kcal/mol) compared to the best-docked candidate (**15**) with a score of −6.8508 Kcal/mol. The standard, doxorubicin had a binding affinity score of −4.3607 Kcal/mol. These results indicated that the compound **15** would have better binding with estrogen receptor-beta (ERbeta) (pdb id: 1x7b) compared to others. This trend for the best-docked and least docked candidates matched with the actual in vitro data in the MCF-7 cell line, suggesting a plausible inhibitory target for synthesized molecules. 

**Figure 3:**
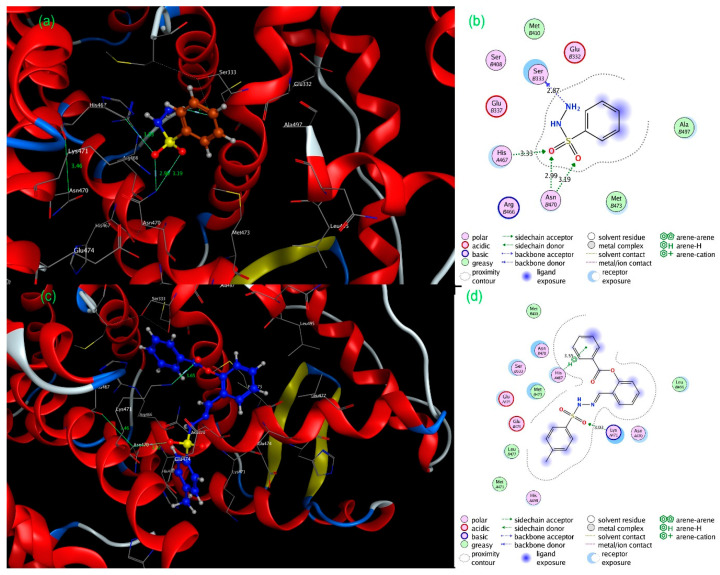
Three-dimensional (**a**,**c**) and two-dimensional (**b**,**d**) interaction diagrams for the least docked molecule (**1**) and best-docked molecule (**15**) inside the binding pocket of target 1x7b.

**Figure 4:**
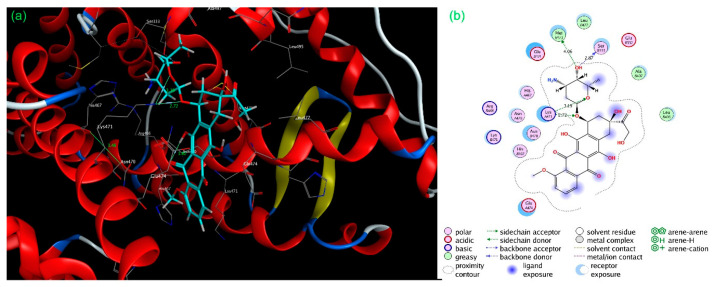
Three-dimensional (**a**) and two-dimensional (**b**) interaction diagram for the standard, doxorubicin inside the binding pocket of target 1x7b.

The least docked candidate (**1**) had interactions with amino acid residues such as SerB333 (distance: 2.87 Å), HisA467 (distance: 3.33 Å), and AsnB470 (distance: 2.99 Å) via backbone acceptor, sidechain acceptor and H-bonding, respectively (Figure 3a,b). The best-docked molecule (**15**) had two interactions, one with HisA467 (distance: 3.55 Å) as an Arene—---H hydrophobic type and a side chain donor type with LysA471 (distance: 3.03 Å) (Figure 3c,d). Some polar amino acid residues were also observed, such as AsnB470, SerB333, HisA498, AsnA470, GluB474, and HisA467. For the standard doxorubicin, we noticed varied interactions with amino acid residues such as MetB473, SerB333, and LysA471, among others (Figure 4a,b). These interactions are of the following types: side chain donor (OH----MetB473) (distance: 4.06 Å); OH----SerB333 (backbone acceptor, H-Bonding) (distance: 2.87 Å); and -C-O-C----LysA471 (side chain acceptor) (distance: 2.72 Å). (S)-Equol, a non-steroidal estrogen agonist, is a soy-derived isoflavonoid molecule produced by the metabolism of the isoflavone daidzein by the intestinal flora. (S)-Equol is a selective estrogen receptor β (ERβ) agonist. The (S)-equol had similar binding residues to the rest of the binding molecules; however, its binding affinity (docking score) remained around −3.76 Kcal/mol (K_d_ = 1.74 × 10^−3^ M). Higher (more negative) docking scores for synthetic molecules suggested that they are antagonists that competitively displace agonists at the receptor. We also docked the positive control, Estrogen, which is a naturally occurring ‘tight binder’, to said receptor and observed that it had a K_d_ value of 5.18 × 10^−5^ M (low K_d_ value than most of compounds, synthesized). However, molecule **15** had a lower K_d_ value than the agonist itself, indicating a stronger binding of (**15**) to the receptor than estrogen.

Based on our initial observations, these compounds exhibit significant interactions between ligands and receptors. Table 2 shows the interacting residues for synthesized compounds along with their docking scores.

### 2.4. In Silico ADME Studies

For all currently synthesized compounds, we screened them for theoretical pharmacokinetic calculations using the ‘SwissADME’ online platform available at: [http://www.swissadme.ch/index.php, accessed on 12 March 2024]. It was observed that the majority of compounds followed the Lipinskis’ rule of five (except molecule (**17**)). Furthermore, except molecules **1**, **2** and **19**, compounds exhibited inhibitions against the CYP enzymes such as CYP2C19, CYP2C9 and CYP1A2. The partition coefficient values (LogP) were within acceptable limits (lipophilicity values less than 4). We observed mixed gastrointestinal passive absorption profiles for all the compounds. The majority of compounds did not cross the BBB (blood–brain barrier). Table 3 lists various pharmacokinetic parameters. Overall, we observed good pharmacokinetic profiles for the synthesized candidates.

## 3. Materials and Methods

### 3.1. General

All chemicals required to synthesize the targeted compounds and conduct anticancer activity studies were purchased from Sigma-Aldrich (St. Louis, MO, USA), Merck (Darmstadt, Germany), and Alfa Aesar (Haverhill, MA, USA), and they were employed without further purification. The progress of the reactions and the purity of the synthesized chemicals were confirmed using Merck aluminum TLC plates with 0.2 mm silica gel 60 F-254 (Merck, Darmstadt, Germany). The melting points of all synthesized compounds were determined using a DMP-100 (AELab, Guangzhou, China) digital melting points apparatus. The structures of the targeted compounds were characterized by FT-IR (Cary 630 FT-IR spectrometer, Agilent, Tokyo, Japan), ^1^H- and ^13^C-NMR (Bruker Avance III 400 MHz spectrometer, Bruker, Ettlingen. Germany), and elemental analysis (Thermo Scientific Flash 2000 CHNS elemental analyzer, Thermo Scientific, Waltham, MA, USA).

#### 3.1.1. General Procedures for the Synthesis of Sulfonyl Hydrazides (**1** and **2**)

In a 50 mL round-bottomed flask, benzenesulfonyl chloride (or *p*-toluenesulfonyl chloride) (5 mmol) was dissolved in 10 mL of tetrahydrofuran. The temperature of the solution was lowered to 0 °C using an ice bath. After adding hydrazine monohydrate (5 mmol) dropwise to this solution, the solution was stirred at °C for 1 h with constant stirring [33]. Reaction progress was monitored by TLC. The solvent was then evaporated under reduced pressure. The crude product obtained was collected through filtration, washed with water, and recrystallized from ethanol to afford benzenesulfonyl hydrazide or *p*-toluenesulfonyl hydrazide.

*Benzensulfonyl hydrazide* (**1**)

White solid, yield: 91%, m.p.: 101–103 °C (Lit. [47] 102–104 °C). 

*4-Methylbenzensulfonyl hydrazide* (**2**)

White solid, yield: 93%, m.p.: 104–106 °C (Lit. [48] 104–107 °C). 

#### 3.1.2. General Procedures for the Synthesis of Benzoates (**3**–**8**)

Compounds **3**–**8** were synthesized in our previous studies [34,35]. A solution of an appropriate phenolic aldehyde (5.0 mmol) in pyridine (10 mL) in a 50 mL round-bottomed flask was slowly added dropwise to a solution of a suitable benzoyl chloride derivative (5.0 mmol) in 10 mL of pyridine [35]. The resulting mixture and stirred under reflux at 120 °C for 1 h with constant stirring. After cooling the reaction mixture to room temperature, it was poured into a beaker containing 50 mL of crushed ice. The esterified product was collected by filtration and washed with excess distilled water (100 mL). Finally, the residue was recrystallized from ethanol to yield the desired benzoate derivative.

#### 3.1.3. General Methods for the Synthesis of the Sulfonyl Hydrazones (**9**–**20**)

A solution of an appropriate hydrazide (2 mmol) and an aryl ester derivative (2 mmol) dissolved in 10 mL of absolute ethanol in a 50 mL round-bottomed flask was stirred under reflux for 4 h with constant stirring [33]. The progress of the reaction was monitored by TLC using a solvent system consisting of *n*-hexane and ethyl acetate (1:2). Upon the completion of the reaction, the mixture was cooled to room temperature, resulting in the formation of a precipitate. The precipitate was collected by filtration, washed with petroleum ether, and purified by ethanol crystallization to give the desired target compounds. 

*N′-(2-{[Benzoyl]oxy}benzylidene)benzenesulfonohydrazide* (**9**)

White solid, yield: 77%, m.p.: 191–192 °C. FT-IR/ATR (cm^−1^), υ_max_: 3187 (N-H), 3064 (C-H), 1733 (C=O), 1600 (C=N), 1319 and 1165 (SO_2_). ^1^H NMR (400 MHz, DMSO-*d*_6_), ppm: δ 11.58 (s, 1H, –SO_2_N*H*–), 8.13 (d, *J* = 7.7 Hz, 2H, Ar-*H*), 8.02 (s, 1H, –C*H*=N–), 7.82–7.72 (m, 4H, Ar-*H*), 7.68–7.53 (m, 5H, Ar-*H*), 7.48 (t, *J* = 7.7 Hz, 1H, Ar-*H*), and 7.38–7.28 (m, 2H, Ar-*H*). ^13^C NMR (100 MHz, DMSO-*d*_6_), ppm: δ 164.88 (*C*=O), 149.41 (*C*=N), 141.90, 139.35, 134.74, 133.57, 131.66, 130.47 (2C), 129.73 (2C), 129.46 (2C), 128.79, 127.51 (2C), 127.08, 126.51, 126.45, and 123.89 (Ar-*C*). Anal. Calcd. for C_20_H_16_N_2_O_4_S: C, 63.15; H, 4.24; N, 7.36; S, 8.43%. Found: C, 63.32; H, 4.12; N, 7.53; S, 8.69%.

*N′-(2-{[4-Nitrobenzoyl]oxy}benzylidene)benzenesulfonohydrazide* (**10**)

White solid, yield: 80%, m.p.: 182–183 °C. FT-IR/ATR (cm^−1^), υ_max_: 3180 (N-H), 3074 (C-H), 1744 (C=O), 1602 (C=N), 1519 and 1347 (NO_2_), 1325 and 1161 (SO_2_). ^1^H NMR (400 MHz, DMSO-*d*_6_), ppm: δ 11.57 (s, 1H, –SO_2_N*H*–), 8.41 (d, *J* = 8.8 Hz, 2H, Ar-*H*), 8.34 (d, *J* = 8.7 Hz, 2H, Ar-*H*), 8.01 (s, 1H, –C*H*=N–), 7.78–7.70 (m, 3H, Ar-*H*), 7.62 (d, *J* = 7.2 Hz, 1H, Ar-*H*), 7.56–7.48 (m, 3H, Ar-*H*), and 7.40–7.33 (m, 2H, Ar-*H*). ^13^C NMR (100 MHz, DMSO-*d*_6_), ppm: δ 163.45 (*C*=O), 151.13 (*C*=N), 148.88, 142.39, 139.34, 134.41, 133.53, 132.00 (2C), 131.68, 129.71 (2C), 127.47, 127.42 (2C), 127.37, 126.27, 124.37 (2C), and 123.82 (Ar-*C*). Anal. Calcd. for C_20_H_15_N_3_O_6_S: C, 56.47; H, 3.55; N, 9.88; S, 7.54%. Found: C, 56.55; H, 3.20; N, 9.61; S, 7.83%.

*N′-(2-{[3,5-Dinitrobenzoyl]oxy}benzylidene)benzenesulfonohydrazide* (**11**)

White solid, yield: 73%, m.p.: 189–190 °C. FT-IR/ATR (cm^−1^), υ_max_: 3206 (N-H), 3086 (C-H), 1737 (C=O), 1627 (C=N), 1547 and 1345 (NO_2_), 1326 and 1174 (SO_2_). ^1^H NMR (400 MHz, DMSO-*d*_6_), ppm: δ 11.61 (s, 1H, –SO_2_N*H*–), 9.14 (t, *J* = 2.1 Hz, 1H, Ar-*H*), 8.99 (d, *J* = 2.1 Hz, 2H, Ar-*H*), 8.01 (s, 1H, –C*H*=N–), 7.70 (d, *J* = 7.7 Hz, 1H, Ar-*H*), 7.63–7.45 (m, 6H, Ar-*H*), 7.42 (d, *J* = 7.6 Hz, 1H, Ar-*H*), and 7.38 (d, *J* = 8.0 Hz, 1H, Ar-*H*). ^13^C NMR (100 MHz, DMSO-*d*_6_), ppm: δ 161.79 (C=O), 148.15 (C=N), 148.73 (2C), 143.72, 139.42, 133.40, 132.22, 131.70, 130.10 (2C), 129.61 (2C), 129.49, 127.66, 127.05 (2C), 126.10, 123.93, and 123.61 (Ar-*C*). Anal. Calcd. for C_20_H_14_N_4_O_8_S: C, 51.07; H, 3.00; N, 11.91; S, 6.82%. Found: C, 51.13; H, 3.11; N, 11.75; S, 6.49%.

*N′-(4-{[Benzoyl]oxy}benzylidene)benzenesulfonohydrazide* (**12)**

White solid, yield: 72%, m.p.: 199–200 °C. FT-IR/ATR (cm^−1^), υ_max_: 3189 (N-H), 3067 (C-H), 1706 (C=O), 1597 (C=N), 1325 and 1158 (SO_2_). ^1^H NMR (400 MHz, DMSO-*d*_6_), ppm: δ 11.57 (s, 1H, –SO_2_N*H*–), 8.11 (d, *J* = 7.5 Hz, 2H, Ar-*H*), 7.96 (s, 1H, –C*H*=N–), 7.90 (d, *J* = 6.7 Hz, 2H, Ar-*H*), 7.74 (d, *J* = 7.5 Hz, 1H, Ar-*H*), 7.66–7.58 (m, 7H, Ar-*H*), and 7.31 (d, *J* = 8.5 Hz, 2H, Ar-*H*). ^13^C NMR (100 MHz, DMSO-*d*_6_), ppm: δ 164.82 (*C*=O), 152.29 (*C*=N), 146.73, 139.46, 134.59, 133.52, 131.95, 130.27 (2C), 129.71 (2C), 129.43 (2C), 129.15, 128.47 (2C), 127.63 (2C), and 122.90 (2C) (Ar-*C*). Anal. Calcd. for C_20_H_16_N_2_O_4_S: C, 63.15; H, 4.24; N, 7.36; S, 8.43%. Found: C, 63.24; H, 4.33; N, 7.17; S, 8.71%.

*N′-(4-{[4-Nitrobenzoyl]oxy}benzylidene)benzenesulfonohydrazide* (**13**)

White solid, yield: 81%, m.p.: 205–206 °C. FT-IR/ATR (cm^−1^), υ_max_: 3172 (N-H), 3078 (C-H), 1740 (C=O), 1601 (C=N), 1522 and 1344 (NO_2_), 1324 and 1160 (SO_2_). ^1^H NMR (400 MHz, DMSO-*d*_6_), ppm: δ 11.58 (s, 1H, –SO_2_N*H*–), 8.39 (d, *J* = 8.7 Hz, 2H, Ar-*H*), 8.34 (d, *J* = 8.8 Hz, 2H, Ar-*H*), 7.96 (s, 1H, –C*H*=N–), 7.89 (d, *J* = 6.8 Hz, 2H, Ar-*H*), 7.69–7.58 (m, 5H, Ar-*H*), and 7.36 (d, *J* = 8.5 Hz, 2H, Ar-*H*). ^13^C NMR (100 MHz, DMSO-*d*_6_), ppm: δ 163.43 (*C*=O), 152.00 (*C*=N), 151.07, 146.63, 139.44, 134.73, 133.54, 132.25, 131.79 (2C), 129.72 (2C), 128.52 (2C), 127.62 (2C), 124.45 (2C), and 122.79 (2C) (Ar-*C*). Anal. Calcd. for C_20_H_15_N_3_O_6_S: C, 56.47; H, 3.55; N, 9.88; S, 7.54%. Found: C, 56.70; H, 3.62; N, 9.45; S, 7.19%.

*N′-(4-{[3,5-Dinitrobenzoyl]oxy}benzylidene)benzenesulfonohydrazide* (**14**)

White solid, yield: 74%, m.p.: 222–223 °C. FT-IR/ATR (cm^−1^), υ_max_: 3243 (N-H), 3104 (C-H), 1743 (C=O), 1599 (C=N), 1540 and 1367 (NO_2_), 1342 and 1167 (SO_2_). ^1^H NMR (400 MHz, DMSO-*d*_6_), ppm: δ 11.59 (s, 1H, –SO_2_N*H*–), 9.11–9.07 (m, 1H, Ar-*H*), 9.05 (d, *J* = 2.0 Hz, 2H, Ar-*H*), 7.96 (s, 1H, –C*H*=N–), 7.88 (d, *J* = 6.8 Hz, 2H, Ar-*H*), 7.69 (d, *J* = 8.6 Hz, 2H, Ar-*H*), 7.66–7.58 (m, 3H, Ar-*H*), and 7.41 (d, *J* = 8.6 Hz, 2H, Ar-*H*). ^13^C NMR (100 MHz, DMSO-*d*_6_), ppm: δ 161.80 (*C*=O), 151.76 (*C*=N), 148.84 (2C), 146.53, 139.44, 133.54, 132.51, 132.48, 129.86 (2C), 129.72 (2C), 128.55 (2C), 127.61(2C), 123.54, and 122.69 (2C) (Ar-*C*). Anal. Calcd. for C_20_H_14_N_4_O_8_S: C, 51.07; H, 3.00; N, 11.91; S, 6.82%. Found: C, 51.21; H, 3.17; N, 12.03; S, 6.95%.

*N′-(2-{[Benzoyl]oxy}benzylidene)-4-methylbenzenesulfonohydrazide* (**15**)

White solid, yield: 76%, m.p.: 194–195 °C. FT-IR/ATR (cm^−1^), υ_max_: 3228 (N-H), 3189 (C-H), 1733 (C=O), 1598 (C=N), 1322 and 1163 (SO_2_). ^1^H NMR (400 MHz, DMSO-*d*_6_), ppm: δ 11.48 (s, 1H, –SO_2_N*H*–), 8.13 (d, *J* = 7.3 Hz, 2H, Ar-*H*), 8.01 (s, 1H, –C*H*=N–), 7.77 (t, *J* = 7.6 Hz, 2H, Ar-*H*), 7.68–7.60 (m, 4H, Ar-*H*), 7.48 (t, *J* = 7.8 Hz, 1H, Ar-*H*), 7.36 (d, *J* = 8.0 Hz, 3H, Ar-*H*), 7.31 (d, *J* = 8.2 Hz, 1H, Ar-*H*), and 2.33 (s, 3H, –C*H*_3_). ^13^C NMR (100 MHz, DMSO-*d*_6_), ppm: δ 164.89 (*C*=O), 149.39 (*C*=N), 144.00, 141.66, 136.47, 134.74, 131.61, 130.47 (2C), 130.16 (2C), 129.46 (2C), 128.79, 127.57 (2C), 127.07, 126.50, 126.45, 123.88 (Ar-*C*), and 21.45 (–*C*H_3_). Anal. Calcd. for C_21_H_18_N_2_O_4_S: C, 63.95; H, 4.60; N, 7.10; S, 8.13%. Found: C, 63.61; H, 4.47; N, 7.36; S, 8.28%.

*N′-(2-{[4-Nitrobenzoyl]oxy}benzylidene)-4-methylbenzenesulfonohydrazide* (**16**)

White solid, yield: 71%, m.p.: 198–200 °C. FT-IR/ATR (cm^−1^), υ_max_: 3271 (N-H), 3074 (C-H), 1741 (C=O), 1600 (C=N), 1519 and 1370 (NO_2_), 1341 and 1158 (SO_2_). ^1^H NMR (400 MHz, DMSO-*d*_6_), ppm: δ 11.47 (s, 1H, –SO_2_N*H*–), 8.41 (d, *J* = 8.9 Hz, 2H, Ar-*H*), 8.34 (d, *J* = 8.8 Hz, 2H, Ar-*H*), 8.00 (s, 1H, –C*H*=N–), 7.78–7.72 (m, 1H, Ar-*H*), 7.61 (d, *J* = 8.2 Hz, 2H, Ar-*H*), 7.50 (t, *J* = 7.7 Hz, 1H, Ar-*H*), 7.40–7.31 (m, 4H, Ar-*H*), and 2.33 (s, 3H, –C*H*_3_). ^13^C NMR (100 MHz, DMSO-*d*_6_), ppm: δ 163.45 (*C*=O), 151.13 (*C*=N), 148.86, 143.95, 142.16, 136.47, 134.41, 131.99 (2C), 131.62, 130.12 (2C), 127.48 (2C), 127.43, 127.36, 126.32, 124.36(2C), 123.81 (Ar-*C*), and 21.44 (–*C*H_3_). Anal. Calcd. for C_21_H_17_N_3_O_6_S: C, 57.40; H, 3.90; N, 9.56; S, 7.30%. Found: C, 57.88; H, 3.68; N, 9.81; S, 7.47%.

*N′-(2-{[3,5-Dinitrobenzoyl]oxy}benzylidene)-4-methylbenzenesulfonohydrazide* (**17**)

White solid, yield: 75%, m.p.: 184–186 °C. FT-IR/ATR (cm^−1^), υ_max_: 3174 (N-H), 3095 (C-H), 1733 (C=O), 1629 (C=N), 1544 and 1364 (NO_2_), 1339 and 1163 (SO_2_). ^1^H NMR (400 MHz, DMSO-*d*_6_), ppm: δ 11.52 (s, 1H, –SO_2_N*H*–), 9.13 (t, *J* = 2.1 Hz, 1H, Ar-*H*), 8.98 (d, *J* = 2.1 Hz, 2H, Ar-*H*), 8.00 (s, 1H, –C*H*=N–), 7.73–7.68 (m, 1H, Ar-*H*), 7.55–7.50 (m, 1H, Ar-*H*), 7.44 (d, *J* = 8.3 Hz, 2H, Ar-*H*), 7.39 (t, *J* = 8.1 Hz, 2H, Ar-*H*), 7.28 (d, *J* = 8.0 Hz, 2H, Ar-*H*), and 2.31 (s, 3H, –C*H*_3_). ^13^C NMR (100 MHz, DMSO-*d*_6_), ppm: δ 161.79 (*C*=O), 148.11 (*C*=N), 148.70 (2C), 143.81, 143.53, 136.59, 132.22, 131.64, 130.08 (2C), 130.02 (2C), 129.53, 127.65, 127.06 (2C), 126.13, 123.92, 123.56 (Ar-*C*), and 21.37 (–*C*H_3_). Anal. Calcd. for C_21_H_16_N_4_O_8_S: C, 52.07; H, 3.33; N, 11.57; S, 6.62%. Found: C, 52.23; H, 3.14; N, 11.79; S, 6.82%.

*N′-(4-{[Benzoyl]oxy}benzylidene)-4-methylbenzenesulfonohydrazide* (**18**)

White solid, yield: 78%, m.p.: 203–204 °C. FT-IR/ATR (cm^−1^), υ_max_: 3220 (N-H), 3069 (C-H), 1708 (C=O), 1595 (C=N), 1325 and 1158 (SO_2_). ^1^H NMR (400 MHz, DMSO-*d*_6_), ppm: δ 11.48 (s, 1H, –SO_2_N*H*–), 8.11 (d, *J* = 7.7 Hz, 2H, Ar-*H*), 7.94 (s, 1H, –C*H*=N–), 7.77 (d, *J* = 8.2 Hz, 2H, Ar-*H*), 7.72 (d, *J* = 7.5 Hz, 1H, Ar-*H*), 7.65 (d, *J* = 8.6 Hz, 2H, Ar-*H*), 7.58 (t, *J* = 7.7 Hz, 2H, Ar-*H*), 7.39 (d, *J* = 8.0 Hz, 2H, Ar-*H*), 7.31 (d, *J* = 8.5 Hz, 2H, Ar-*H*), and 2.34 (s, 3H, –C*H*_3_). ^13^C NMR (100 MHz, DMSO-*d*_6_), ppm: δ 164.84 (*C*=O), 152.26 (*C*=N), 146.52, 143.93, 136.58, 134.60, 132.01, 130.28 (2C), 130.13 (2C), 129.44 (2C), 129.16, 128.43 (2C), 127.68 (2C), 122.90 (2C) (Ar-*C*), and 21.45 (–*C*H_3_). Anal. Calcd. for C_21_H_18_N_2_O_4_S: C, 63.95; H, 4.60; N, 7.10; S, 8.13%. Found: C, 63.79; H, 4.71; N, 7.25; S, 8.03%.

*N′-(4-{[4-Nitrobenzoyl]oxy}benzylidene)-4-methylbenzenesulfonohydrazide* (**19**)

White solid, yield: 70%, m.p.: 191–192 °C. FT-IR/ATR (cm^−1^), υ_max_: 3207 (N-H), 3089 (C-H), 1721 (C=O), 1597 (C=N), 1514 and 1359 (NO_2_), 1325 and 1161 (SO_2_). ^1^H NMR (400 MHz, DMSO-*d*_6_), ppm: δ 11.49 (s, 1H, –SO_2_N*H*–), 8.42–8.30 (m, 4H, Ar-*H*), 7.94 (s, 1H, –C*H*=N–), 7.76 (d, *J* = 8.2 Hz, 2H, Ar-*H*), 7.66 (d, *J* = 8.6 Hz, 2H, Ar-*H*), 7.43–7.33 (m, 4H, Ar-*H*), and 2.34 (s, 3H, –C*H*_3_). ^13^C NMR (100 MHz, DMSO-*d*_6_), ppm: δ 163.43 (*C*=O), 151.96 (*C*=N), 151.06, 146.41, 143.94, 136.57, 134.73, 132.31, 131.78 (2C), 130.14 (2C), 128.48 (2C), 127.68 (2C), 124.44 (2C), 122.77 (2C) (Ar-*C*), and 21.45 (–*C*H_3_). Anal. Calcd. for C_21_H_17_N_3_O_6_S: C, 57.40; H, 3.90; N, 9.56; S, 7.30%. Found: C, 57.64; H, 3.82; N, 9.79; S, 7.58%.

*N′-(4-{[3,5-Dinitrobenzoyl]oxy}benzylidene)-4-methylbenzenesulfonohydrazide* (**20**)

White solid, yield: 77%, m.p.: 200–202 °C. FT-IR/ATR (cm^−1^), υ_max_: 3203 (N-H), 3092 (C-H), 1738 (C=O), 1596 (C=N), 1534 and 1327 (NO_2_), 1327 and 1161 (SO_2_). ^1^H NMR (400 MHz, DMSO-*d*_6_), ppm: δ 11.50 (s, 1H, –SO_2_N*H*–), 9.09 (t, *J* = 2.1 Hz, 1H, Ar-*H*), 9.05 (d, *J* = 2.0 Hz, 2H, Ar-*H*), 7.94 (s, 1H, –C*H*=N–), 7.76 (d, *J* = 8.2 Hz, 2H, Ar-*H*), 7.68 (d, *J* = 8.6 Hz, 2H, Ar-*H*), 7.40 (t, *J* = 7.3 Hz, 4H, Ar-*H*), and 2.35 (s, 3H, –C*H*_3_). ^13^C NMR (100 MHz, DMSO-*d*_6_), ppm: δ 161.81 (*C*=O), 151.73 (*C*=N), 148.84(2C), 146.32, 143.95, 136.55, 132.53, 132.51, 130.14 (2C), 129.85 (2C), 128.52 (2C), 127.66 (2C), 123.54, 122.69 (2C) (Ar-*C*), and 21.45 (–*C*H_3_). Anal. Calcd. for C_21_H_16_N_4_O_8_S: C, 52.07; H, 3.33; N, 11.57; S, 6.62%. Found: C, 52.15; H, 3.48; N, 11.63; S, 6.44%. 

### 3.2. The Cytotoxic Activity Studies

Two cancer cell lines (lung (A549; ATCC CCL-185) and breast (MCF-7; ATCC HTB-22)) were purchased from the American Type Culture Collection (ATCC, Manassas, VA, USA) and cultured in DMEM (for A549)/RPMI (for MCF-7) supplemented with 10% FBS and 1% GlutaMAX. A549 and MCF-7 cells, which were seeded at a density of 5 × 10^3^ cells/well, were exposed to molecules (**1**, **2**, and **9**–**20**) for 72 h [49,50]. Absorbance values at 590 nm were measured using an Epoch 2 ELISA reader (BioTek, Winooski, VT, USA).

### 3.3. Docking Simulations

All the structures were first sketched in ‘ChemBioDraw V 12.0’ and saved in ‘.mol’ files. An X-ray crystal structure of the target protein (PDB IDs 1x7B for MCF-7) was obtained from the RCSB Protein Data Bank [https://www.rcsb.org/structure/1x7b, accessed on 1 June 2024]. Structural defects in these proteins were corrected using MOE software V. 2015 for docking calculations [51]. Default parameters (temperature 300 K, pH 7, solvent 0.1 M, electrostatic energy cut off 15 Å) were applied during calculations [51]. The final docking scores were determined based on the average score of the top 10 docking poses with the lowest binding energy (kcal/mol) for each molecule. A detailed methodology using MOE V. 2015 has been found in the literature [51]. 

### 3.4. In Silico Pharmacokinetics

Understanding the ADME (Absorption, Distribution, Metabolism, and Excretion) properties of drug molecules is crucial in pharmaceutical research [46]. It informs drug development by predicting efficacy, toxicity, and dosing regimens. ADME studies guide the optimization of compounds for better therapeutic outcomes, ensuring safer and more effective medications for patients [46]. The ‘SMILES’ strings for all the synthesized compounds were queries for their theoretical ADME assessments using the well-known online platform ‘SwissADME’ which is available at the following link: (http://www.swissadme.ch/index.php (accessed on 21 May 2024)) [46].

## 4. Conclusions

In this study, new sulfonyl hydrazone derivatives (**9**–**20**) were synthesized for the first time to identify new chemotherapeutic drug candidates for the treatment of lung and breast cancer cell lines. The newly synthesized compounds were characterized using elemental analysis and three spectroscopic methods, and their cytotoxic properties against human lung and breast cancer cell lines at various concentrations for 72 h were determined in vitro and in silico. Among the tested compounds, except compounds **1** and **2**, other compounds (**9**–**20**) showed cytotoxic activity at concentrations lower than 200 µM. It was found that compound **15** showed promising cytotoxic activities against both cell lines with IC_50_ values of 29.59 and 27.70 µM, respectively. The docking simulations revealed that compound **15** exhibited the highest affinity (−6.8508 kcal/mol) for the estrogen receptor-beta (ERbeta) compared to molecule (**1**) (−4.3304 kcal/mol) and doxorubicin (−4.3607 kcal/mol). This suggests that compound **15** is promising for ERβ binding. Most compounds adhered to Lipinski’s rule of five, except molecule (**17**), with acceptable LogP values, indicating favorable lipophilicity. Additionally, some of the compounds demonstrated inhibitory effects on CYP enzymes, except for a few. However, all compounds showed mixed gastrointestinal absorption profiles, with the majority being unable to cross the blood–brain barrier (BBB). Overall, compound **15** emerged as a potential candidate for further investigation due to its modest affinity for ERβ.

## Figures and Tables

**Table 1:** Anticancer activity results of the compounds (**1**, **2**, and **9**–**20**) and cisplatin against cancer cell lines.

Compounds	IC_50_ (µM)	
	A549	MCF-7
**1**	>200	>200
**2**	66.92	>200
**9**	50.72	170.30
**10**	58.83	34.61
**11**	66.47	64.96
**12**	71.80	95.61
**13**	161.10	54.30
**14**	176.70	59.92
**15**	29.59	27.70
**16**	55.21	108.90
**17**	50.91	56.94
**18**	49.89	40.58
**19**	166.20	51.03
**20**	128.30	86.06
**Cisplatin**	22.42	18.01

**Table 2:** The estimated binding affinities of the target compounds for the human estrogen recep-tor β ligand-binding domain (pdb id:1X7B).

Compounds	Molecular Docking Simulations (PDB ID: 1X7B)			(Kd) Values
	Docking Score (Kcal/mol)	RMSD Value (Å)	Residues	
**1**	−4.3304	0.8128	SerB333, AsnB470	6.64 × 10^−4^ M
**2**	−4.4330	0.9198	AsnB470, HisA467, Lys471	5.58 × 10^−4^ M
**9**	−4.8794	1.3110	AsnB470	2.63 × 10^−4^ M
**10**	−6.2329	0.8675	AsnB470, HisA467	2.67 × 10^−5^ M
**11**	−5.7709	1.1250	AsnB470, HisA467, Lys471, Met473	5.82 × 10^−5^ M
**12**	−4.9932	1.7859	Val499, Lys471	2.17 × 10^−4^ M
**13**	−5.5541	1.4146	Met473	8.40 × 10^−5^ M
**14**	−5.0098	1.8998	SerB333	2.11 × 10^−4^ M
**15**	−6.8508	1.6979	His467, Lys471	9.39 × 10^−6^ M
**16**	−4.9910	1.5659	Leu495, met473	2.18 × 10^−4^ M
**17**	−5.7721	1.3471	Lys471, Met473, AsnB470	5.81 × 10^−5^ M
**18**	−5.6341	1.7282	Lys471, Met473, AsnB470	7.34 × 10^−5^ M
**19**	−5.7763	1.6472	Lys471	5.77 × 10^−5^ M
**20**	−5.0043	1.8942	Lys471, Asn470	2.13 × 10^−4^ M
**Doxorubicin**	−4.3607	1.5752	Met473, SerB333, Lys471	6.31 × 10^−4^ M
**Estrogen (Positive Control)**	−5.8400	0.8300	Lys471, Met473, AsnB470	5.18 × 10^−5^ M

**Table 3:** Drug-like Characteristics of Compounds Computed by the Swiss ADME Online Software Program.

Compounds	In Silico Pharmacokinetics Analysis ^a–g^						
	MW (g/mol)	LogP	TPSA	BB	GI Abs	Type of CYP Inhibitions	Rule of Five
**1**	172.20	0.87	80.57	No	High	None	Yes
**2**	186.23	1.11	80.57	No	High	None	Yes
**9**	380.42	2.33	93.21	No	High	CYP2C19, CYP2C9	Yes
**10**	425.41	1.98	139.03	No	Low	CYP2C19, CYP2C9	Yes
**11**	470.41	1.63	184.85	No	Low	CYP2C19, CYP2C9	Yes
**12**	380.42	2.31	93.21	No	High	CYP2C19, CYP2C9	Yes
**13**	425.41	1.84	139.03	No	High	CYP2C19, CYP2C9	Yes
**14**	470.41	1.33	184.85	No	High	CYP2C19, CYP2C9	Yes
**15**	394.44	2.34	93.21	No	High	CYP1A2, CYP2C9	Yes
**16**	439.44	2.61	139.03	No	Low	CYP2C19, CYP2C9	Yes
**17**	484.44	2.33	184.85	No	Low	CYP2C19, CYP2C9	Yes, 1 violation: NorO > 10
**18**	394.44	3.18	93.21	No	High	CYP1A2, CYP2C9	Yes
**19**	439.44	2.45	139.03	No	Low	CYP2C19, CYP2C9	Yes
**20**	484.44	1.58	184.85	No	Low	CYP2C19, CYP2C9	Yes
Doxorubicin	543.52	1.31	206.07 Å	No	Low	None	No

^a^ Molecular weight (recommended value < 500). ^b^ Logarithm of the partition coefficient of the compound between n-octanol and water (recommended value < 5). ^c^ Polar surface area (recommended value ≤ 140 Å^2^). ^d^ Indicates whether the compound passes the BBB or not. ^e^ Degree of gastrointestinal absorption. ^f^ Represents the inhibition of CYP450 subtypes. ^g^ Indicates whether the compound obeys Lipinski’s rule of five or not.

## Data Availability

The original contributions presented in the study are included in the article/Supplementary Material, further inquiries can be directed to the corresponding authors.

## Supplementary Materials

The following supporting information can be downloaded at https://www.mdpi.com/article/10.3390/molecules29153478/s1. The FT-IR, and ^1^H and ^13^C NMR spectra of compounds (**9**–**20**) are available in the Supporting Information file.
