# Supplementary material for: Molecular Hybrid Design, Synthesis, In Vitro Cytotoxicity, In Silico ADME and Molecular Docking Studies of New Benzoate Ester-Linked Arylsulfonyl Hydrazones
Source: Molecules. 2024 Jul 25;29(15):3478. doi: 10.3390/molecules29153478 (PMC11313727; doi:10.3390/molecules29153478)

<u>**Supporting Information**</u>

Molecular Hybrid Design, Synthesis, In Vitro Cytotoxicity, In Silico ADME and Molecular Docking Studies of New Benzoate Ester-Linked Arylsulfonyl Hydrazones

Erdem Ergan [1,*], Reşit Çakmak [2], Eyüp Başaran [3,*], Suraj N. Mali [4], Senem Akkoc [5,6] and Sivakumar Annadurai [7]

[1] Department of Property Protection and Security, Van Security Vocational School, Van Yuzuncu Yil University, Van 65080, Türkiye
[2] Medical Laboratory Techniques Program, Vocational School of Health Services, Batman University, Batman 72060, Türkiye; resit.cakmak@batman.edu.tr
[3] Department of Chemistry and Chemical Processing Technologies, Vocational School of Technical Sciences, Batman University, Batman 72060, Türkiye
[4] School of Pharmacy, D.Y. Patil University (Deemed to be University), Sector 7, Nerul, Navi Mumbai 400706, India; mali.suraj1695@gmail.com
[5] Department of Basic Pharmaceutical Sciences, Faculty of Pharmacy, Suleyman Demirel University, Isparta 32260, Türkiye; senemakkoc@sdu.edu.tr
[6] Faculty of Engineering and Natural Sciences, Bahcesehir University, Istanbul 34353, Türkiye
[7] Department of Pharmacognosy, College of Pharmacy, King Khalid University, Abha 61421, Saudi Arabia; sannadurai@kku.edu.sa
* Correspondence: erdemergan@yyu.edu.tr (E.E.); eyup.basaran@batman.edu.tr (E.B.)

FT-IR spectrum of compound **9**

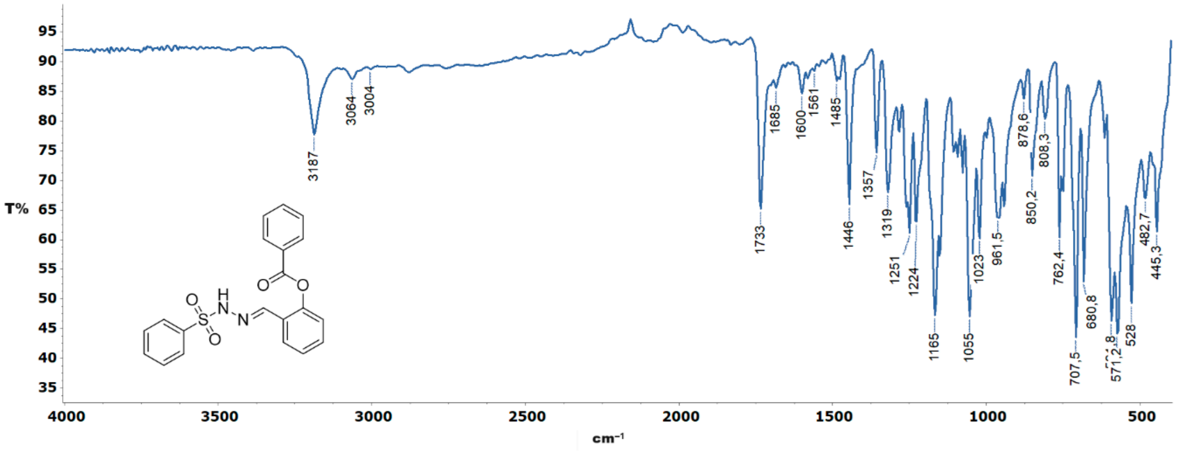

^{1}H NMR spectrum of compound **9**

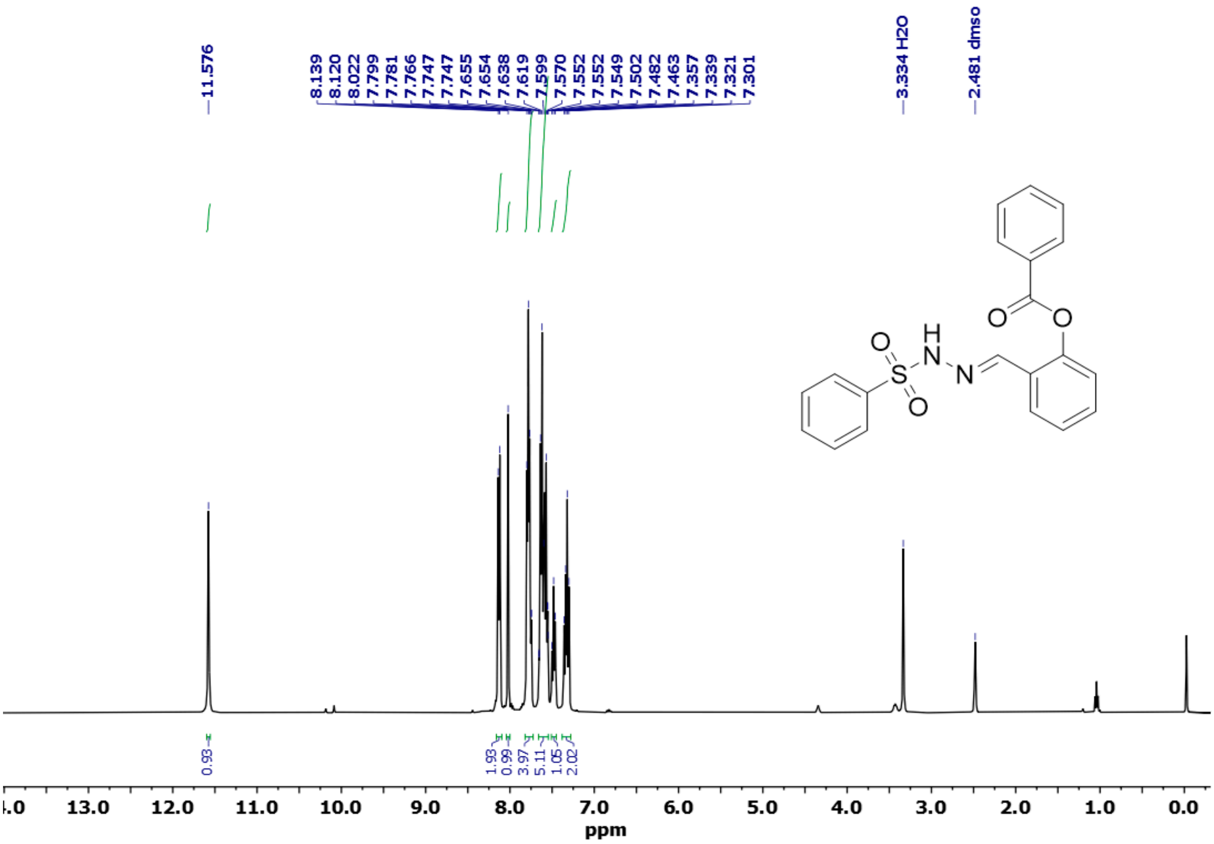

^{13}C NMR spectrum of compound **9**

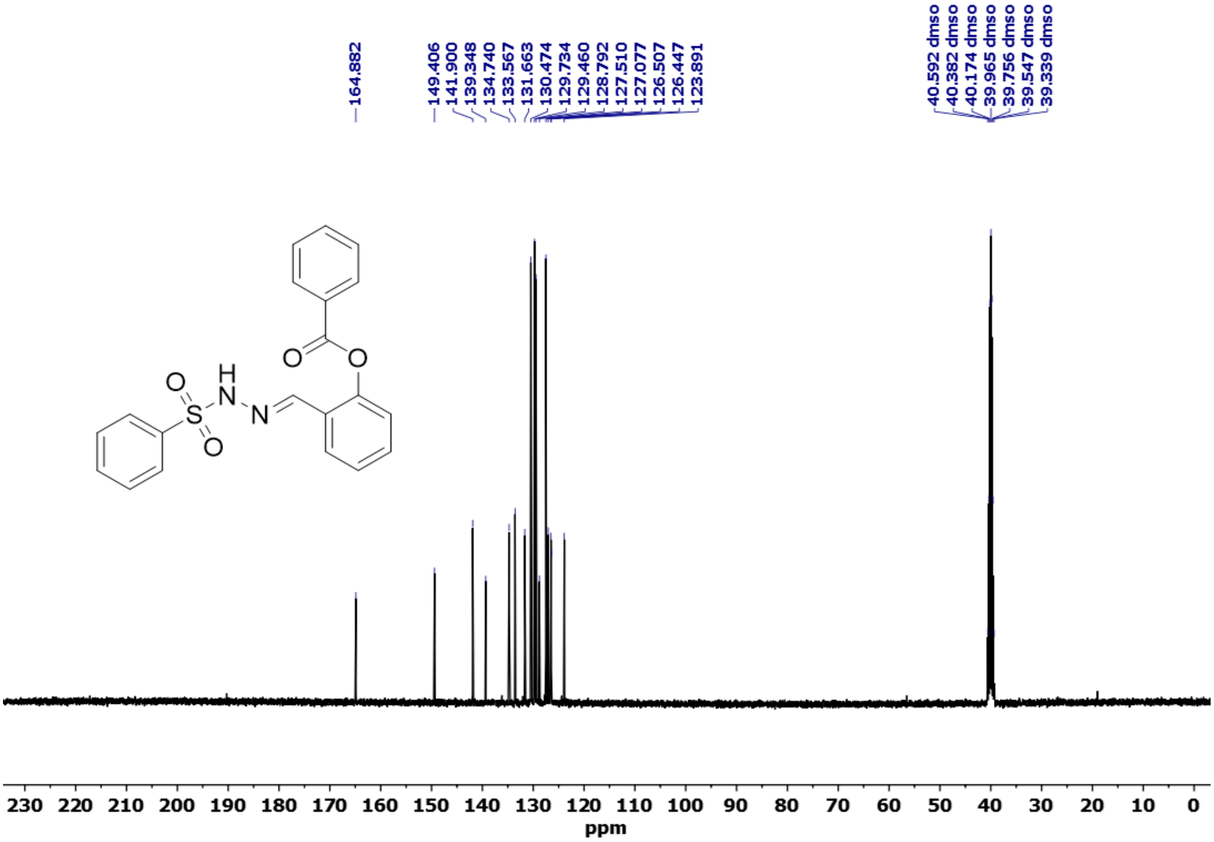

FT-IR spectrum of compound **10**

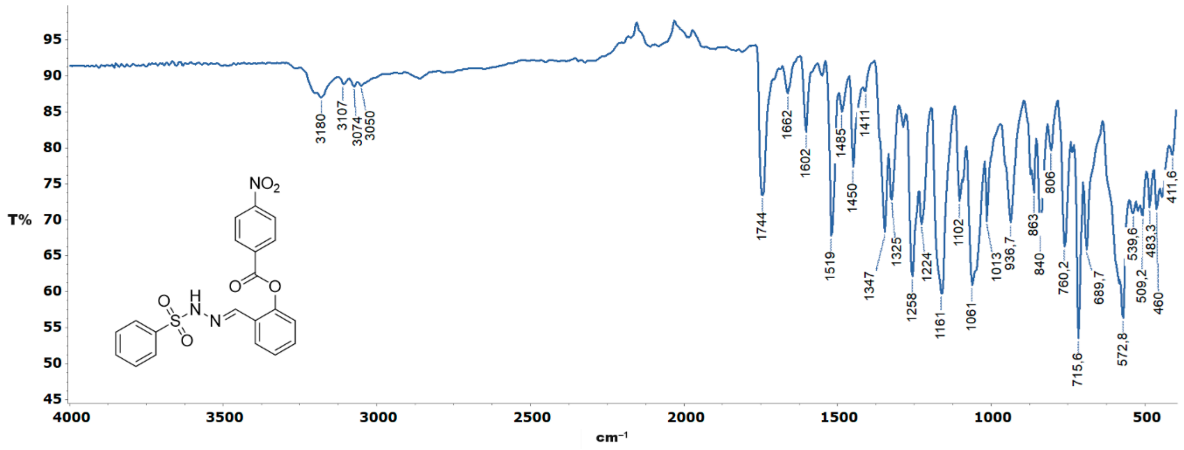

^{1}H NMR spectrum of compound **10**

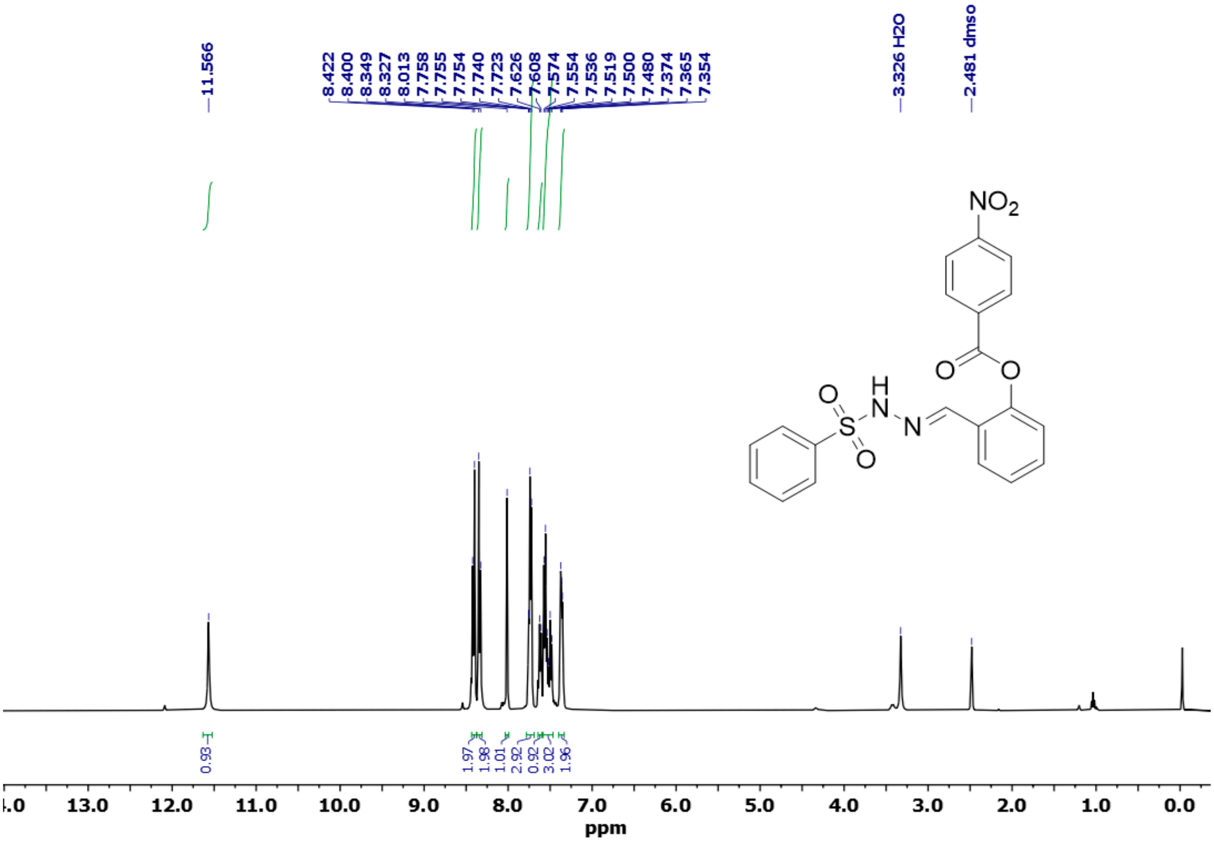

^{13}C NMR spectrum of compound **10**

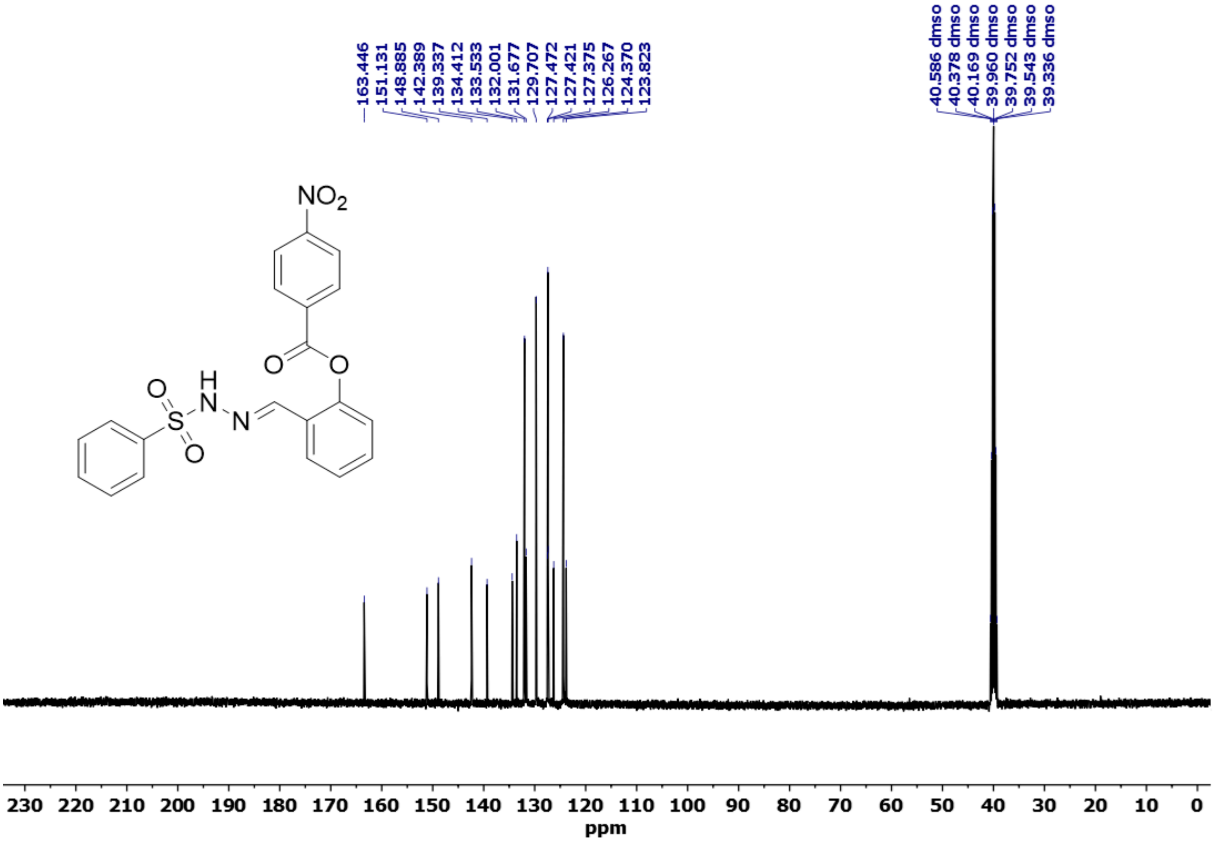

FT-IR spectrum of compound **11**

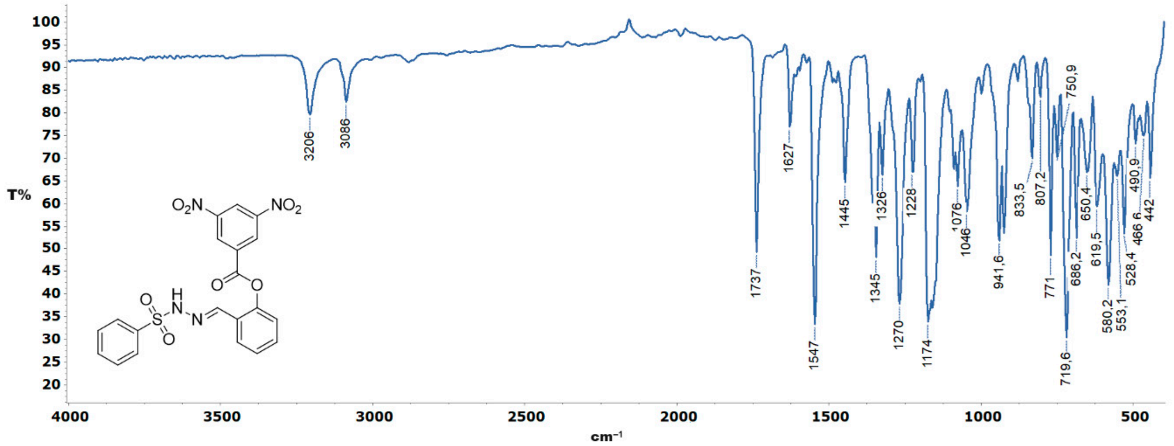

^{1}H NMR spectrum of compound **11**

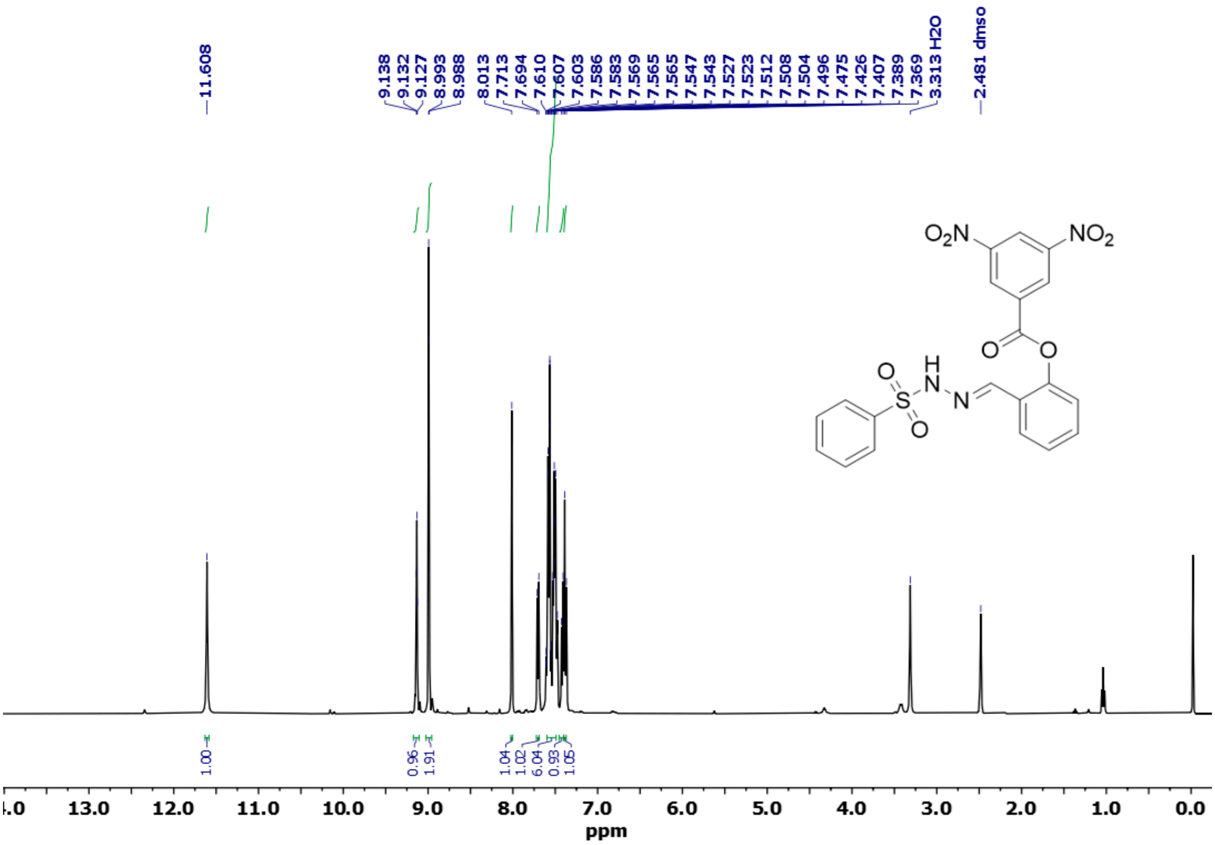

^{13}C NMR spectrum of compound **11**

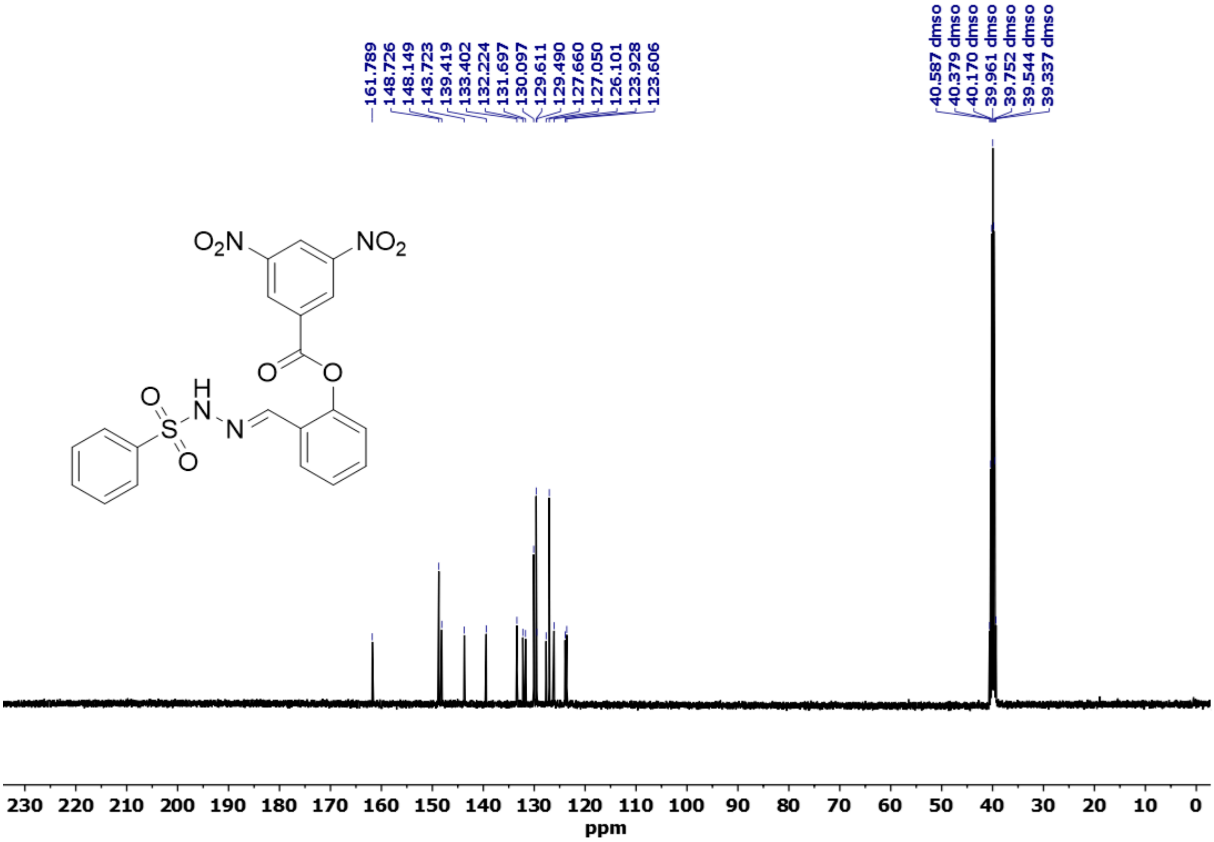

FT-IR spectrum of compound **12**

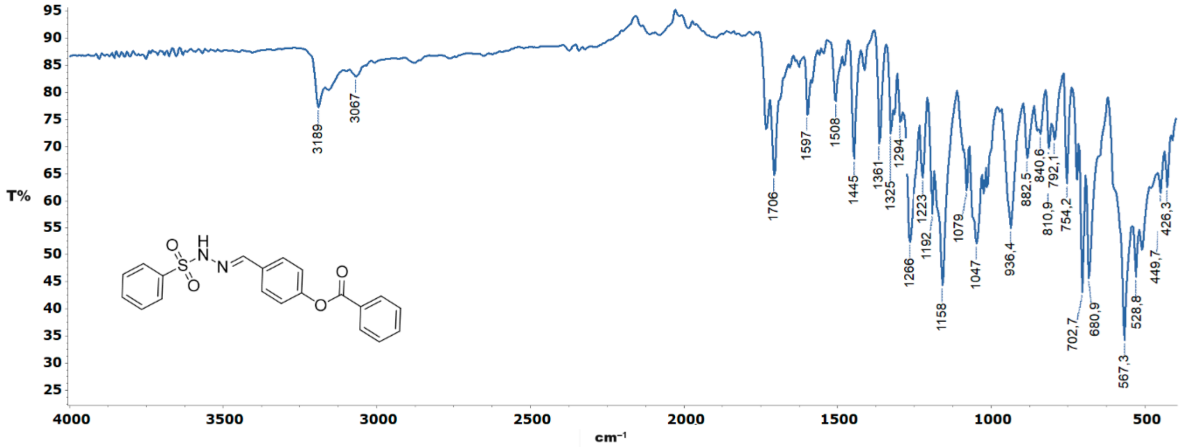

^{1}H NMR spectrum of compound **12**

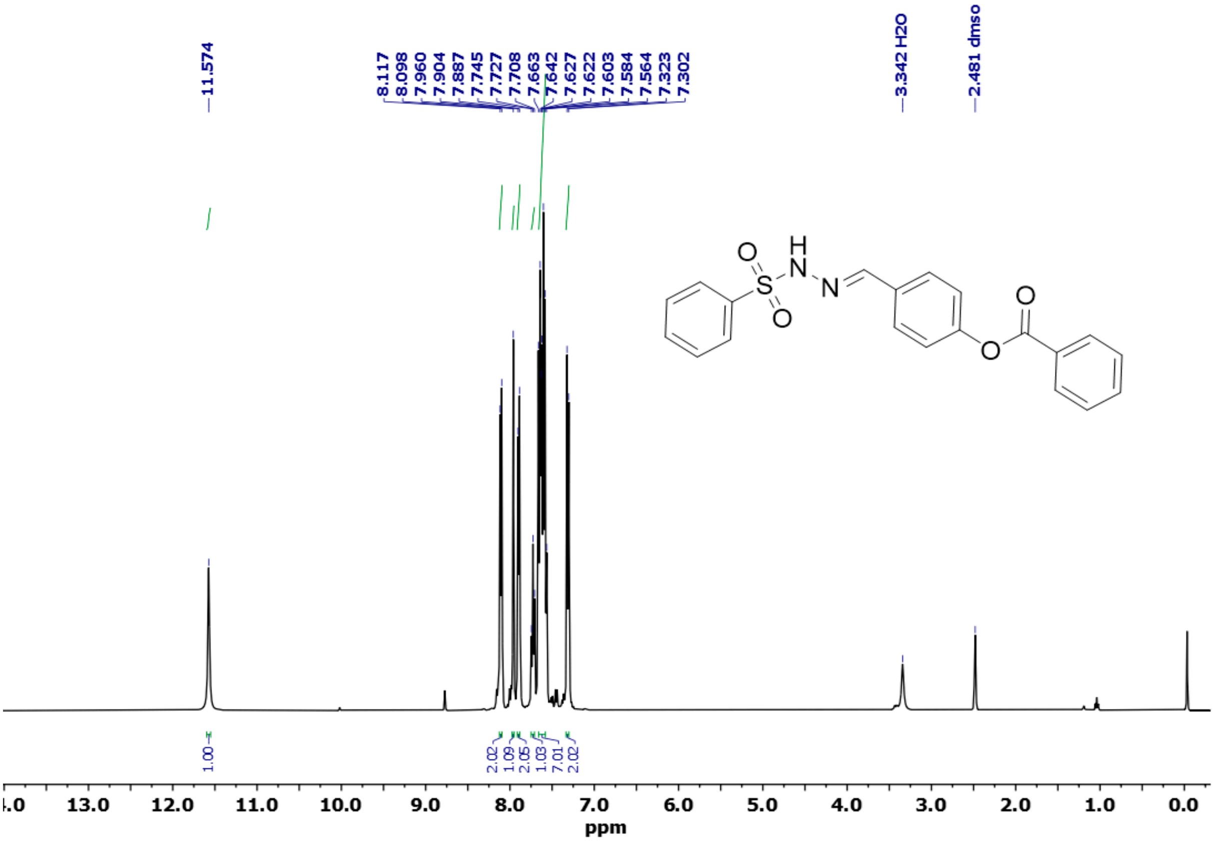

^{13}C NMR spectrum of compound **12**

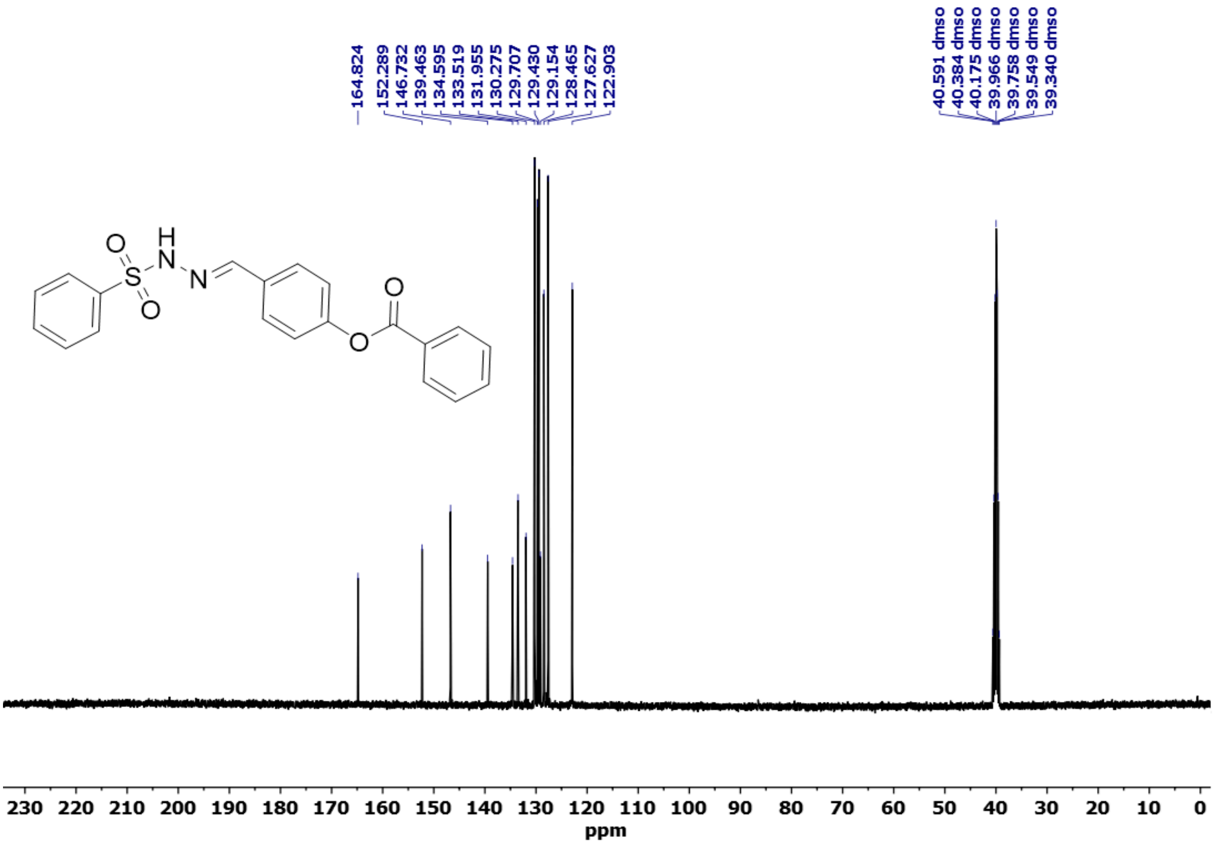

FT-IR spectrum of compound **13**

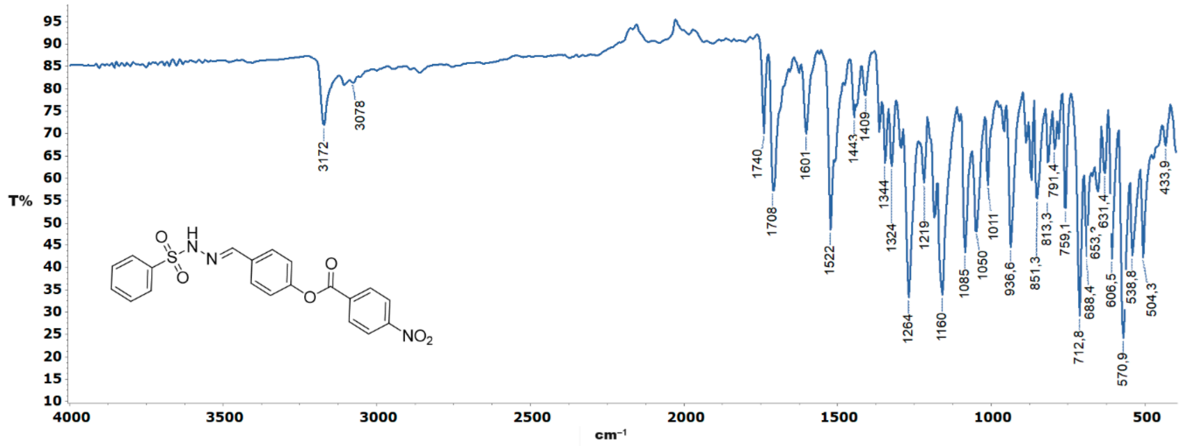

^{1}H NMR spectrum of compound **13**

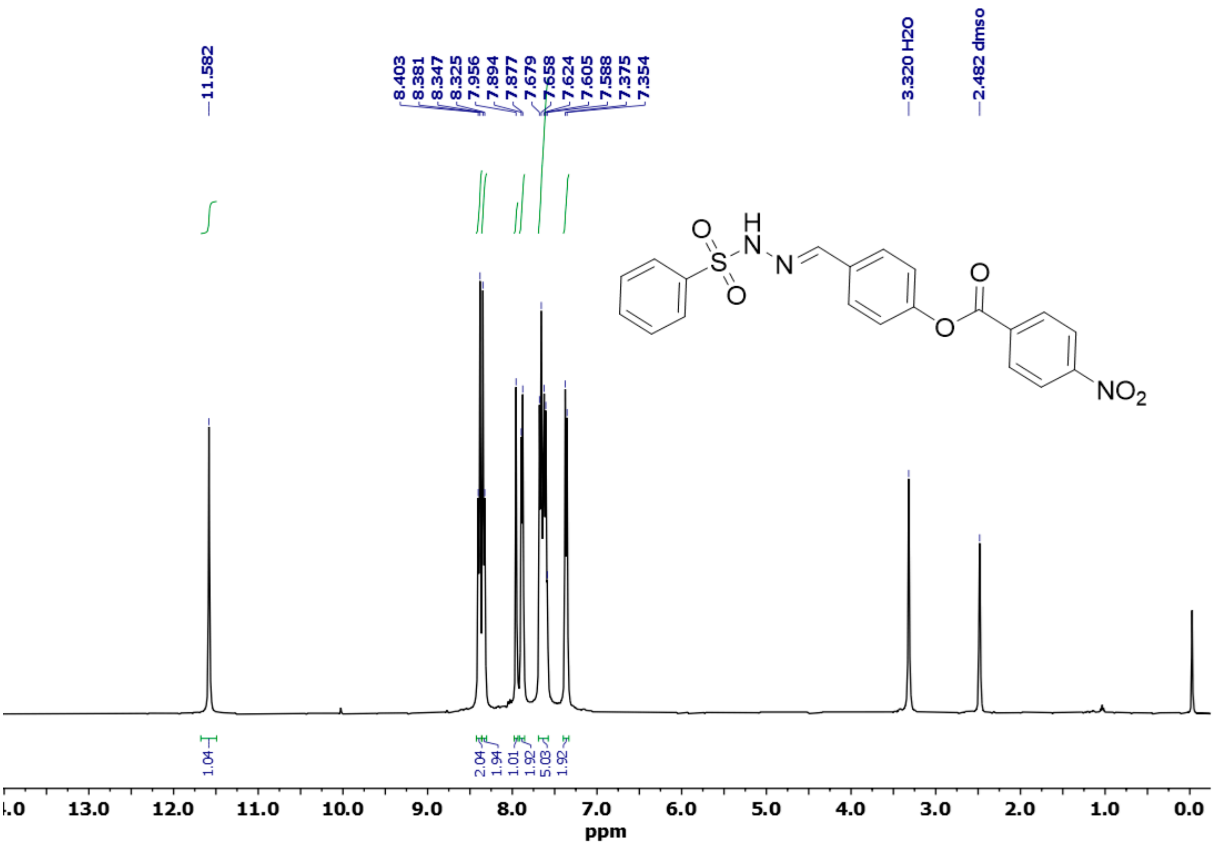

^{13}C NMR spectrum of compound **13**

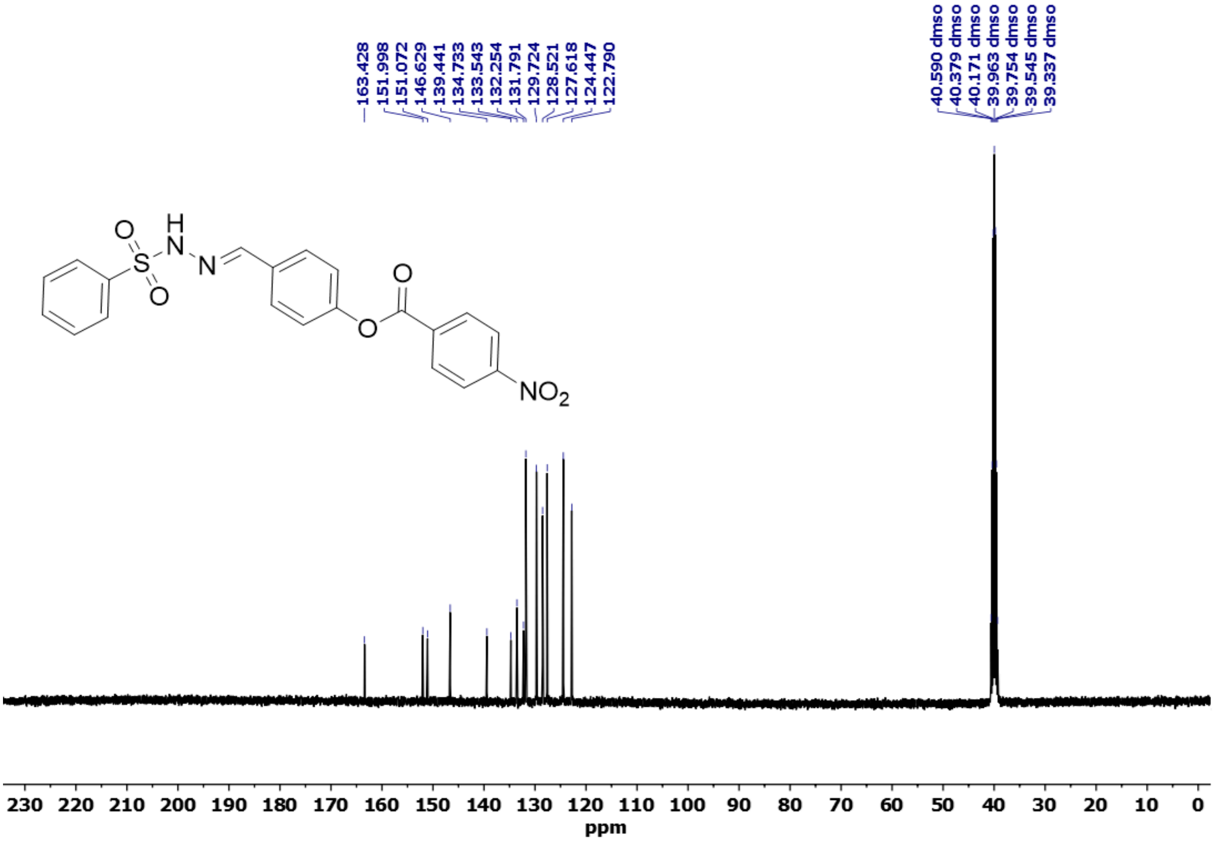

FT-IR spectrum of compound **14**

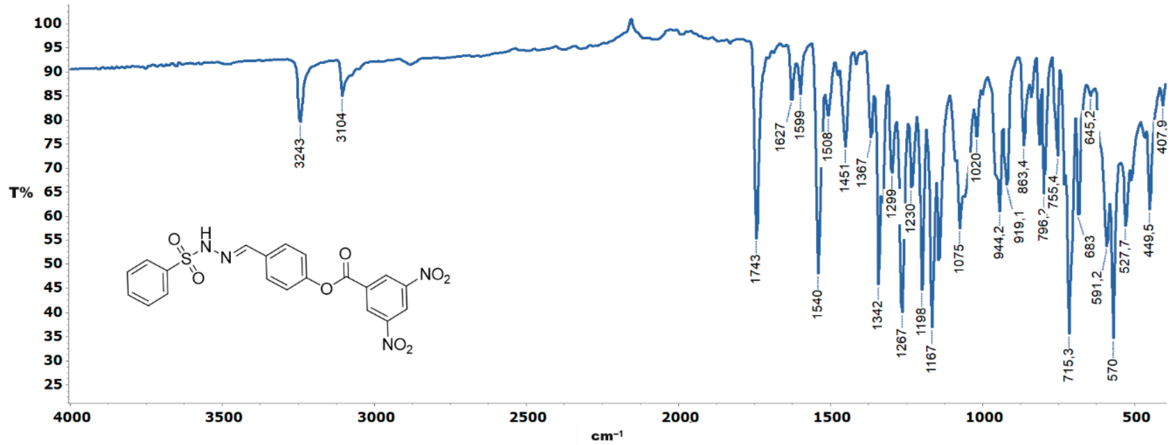

^{1}H NMR spectrum of compound **14**

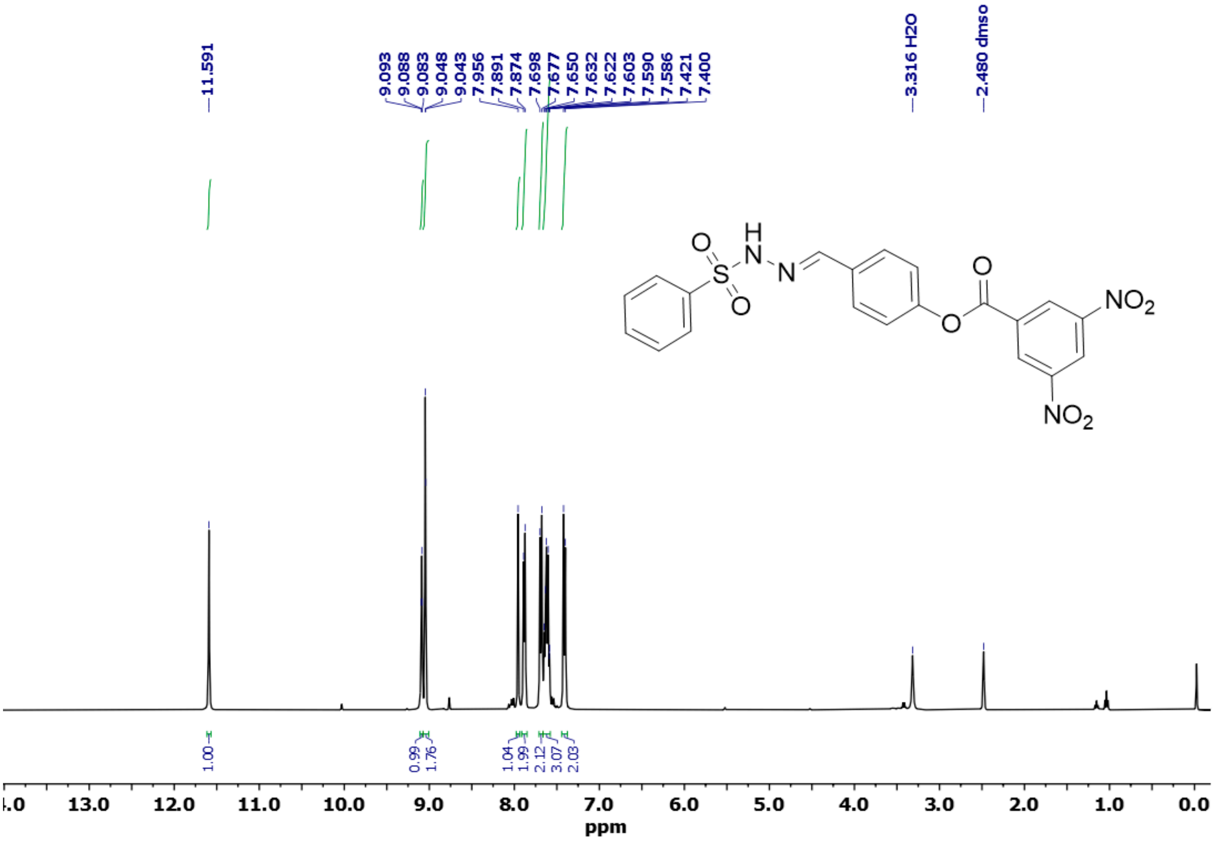

^{13}C NMR spectrum of compound **14**

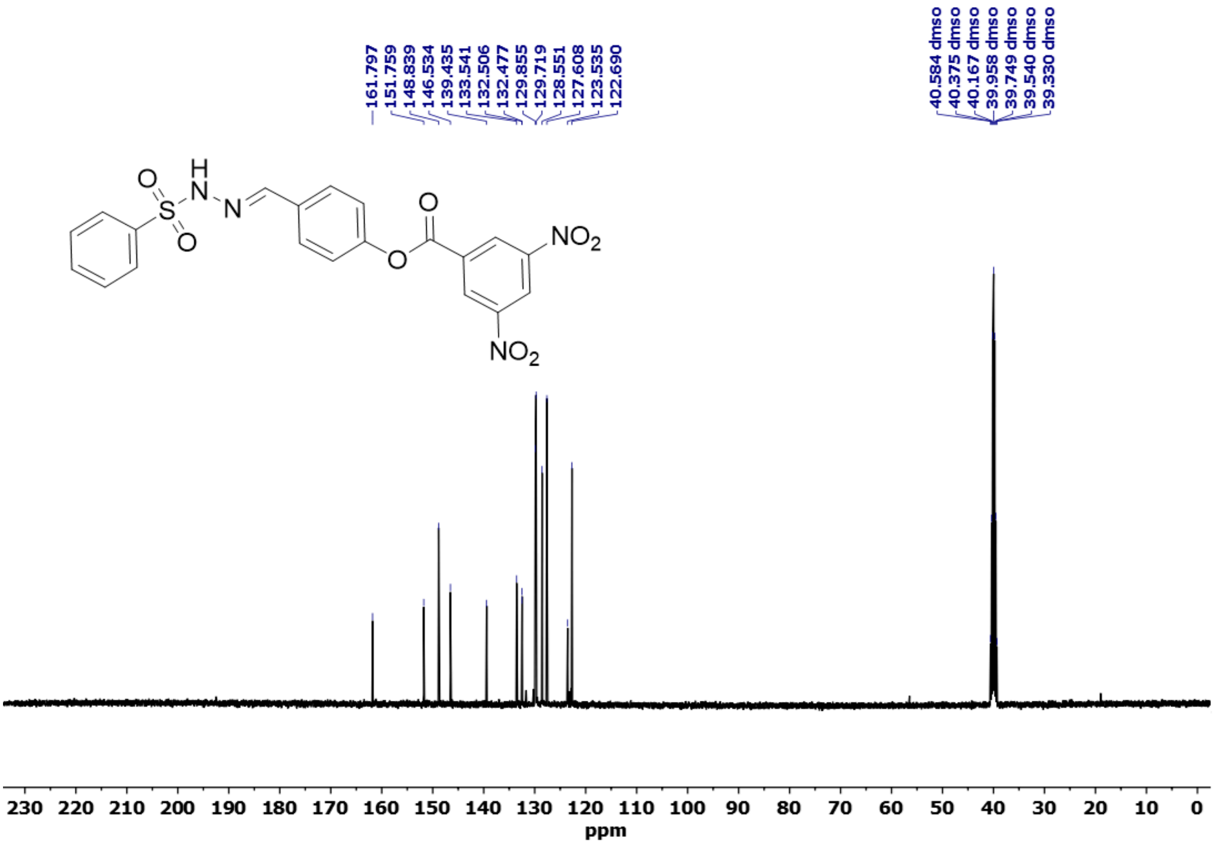

FT-IR spectrum of compound **15**

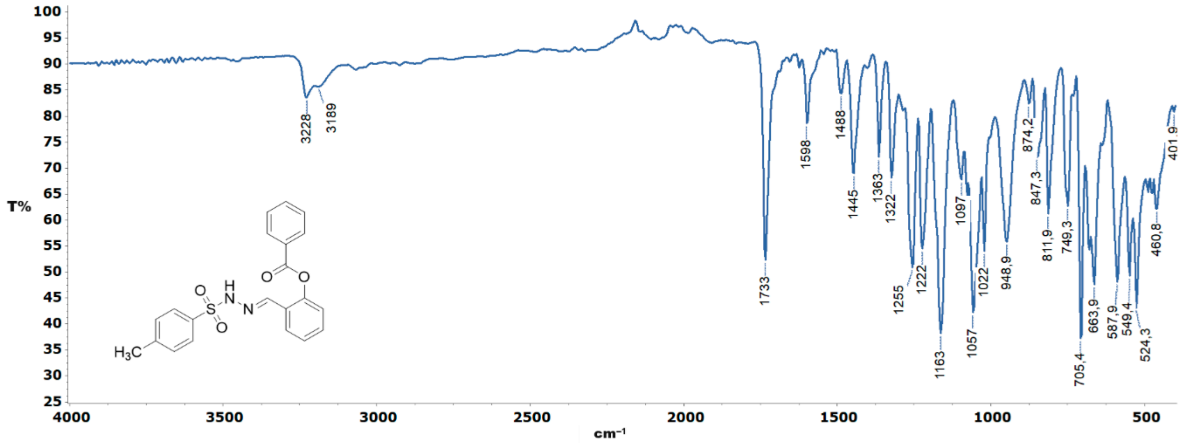

^{1}H NMR spectrum of compound **15**

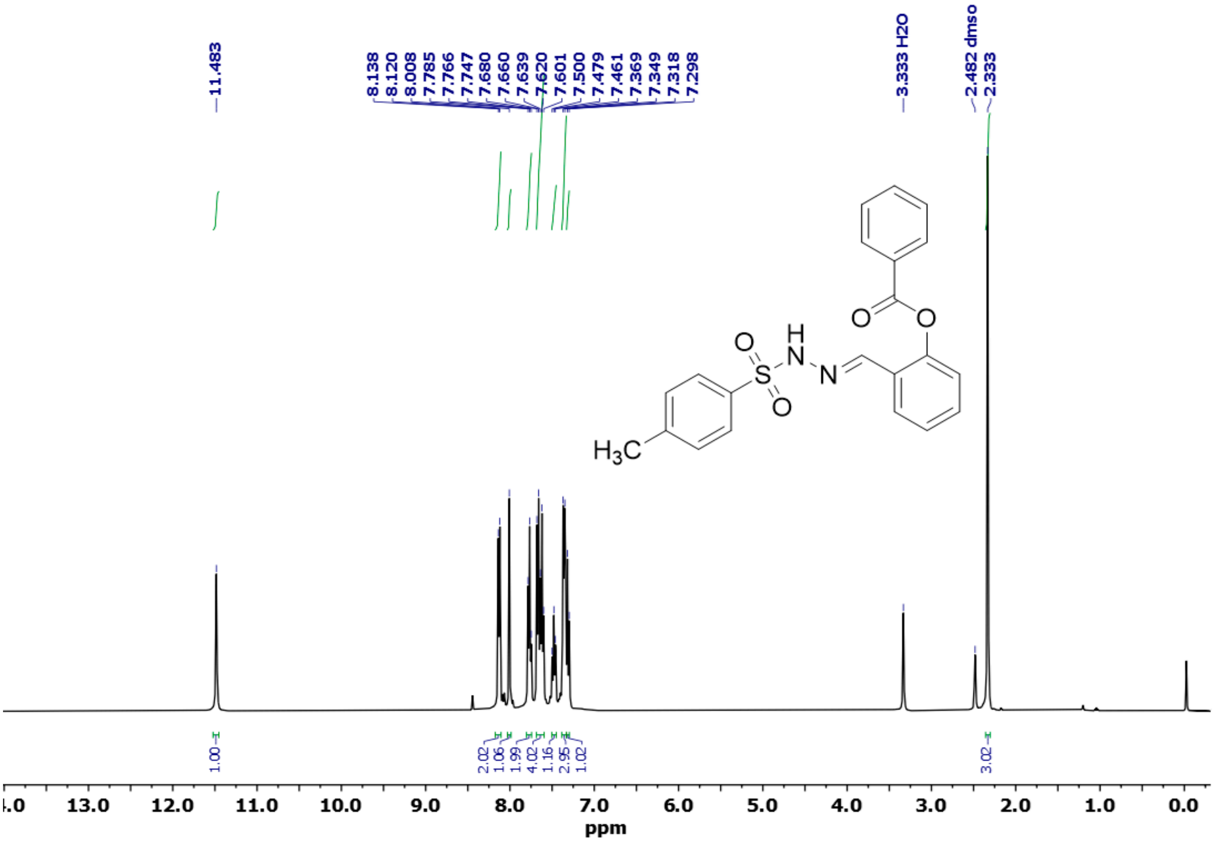

^{13}C NMR spectrum of compound **15**

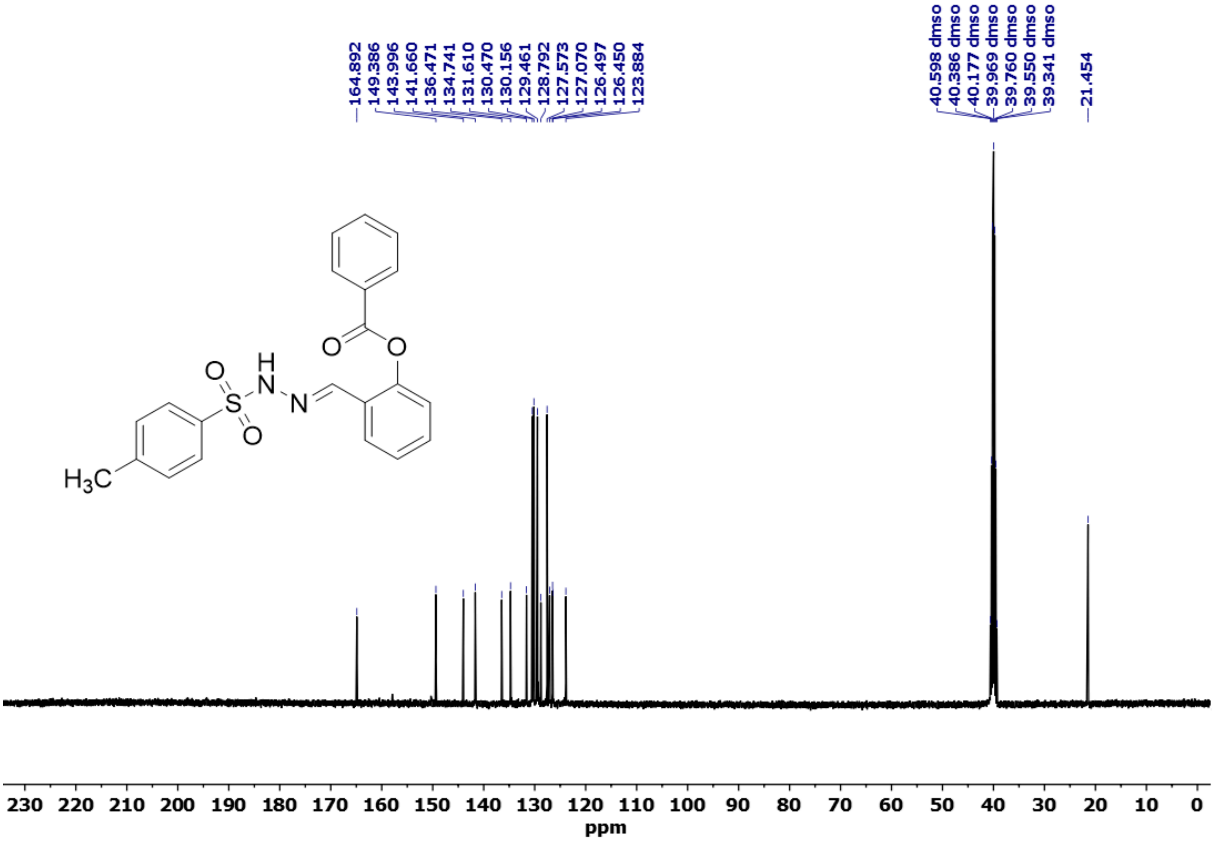

FT-IR spectrum of compound **16**

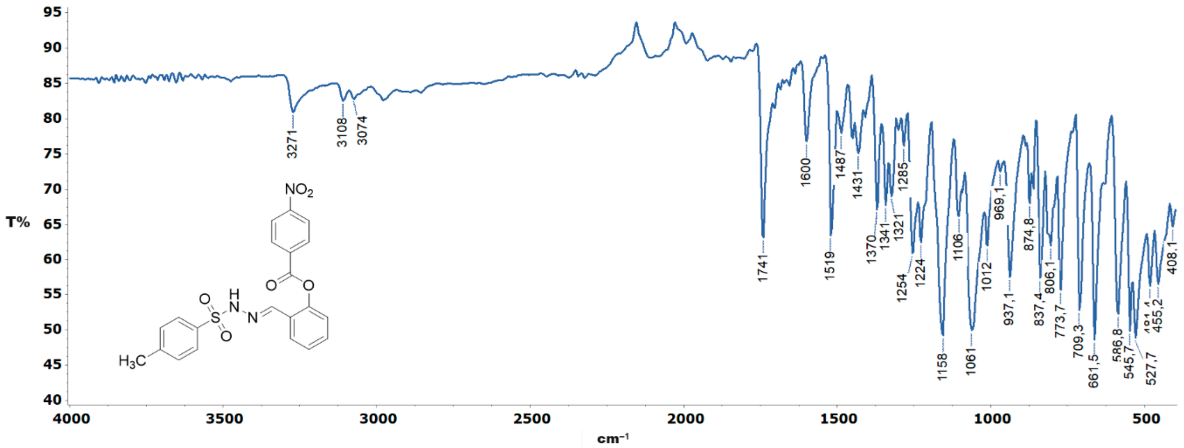

^{1}H NMR spectrum of compound **16**

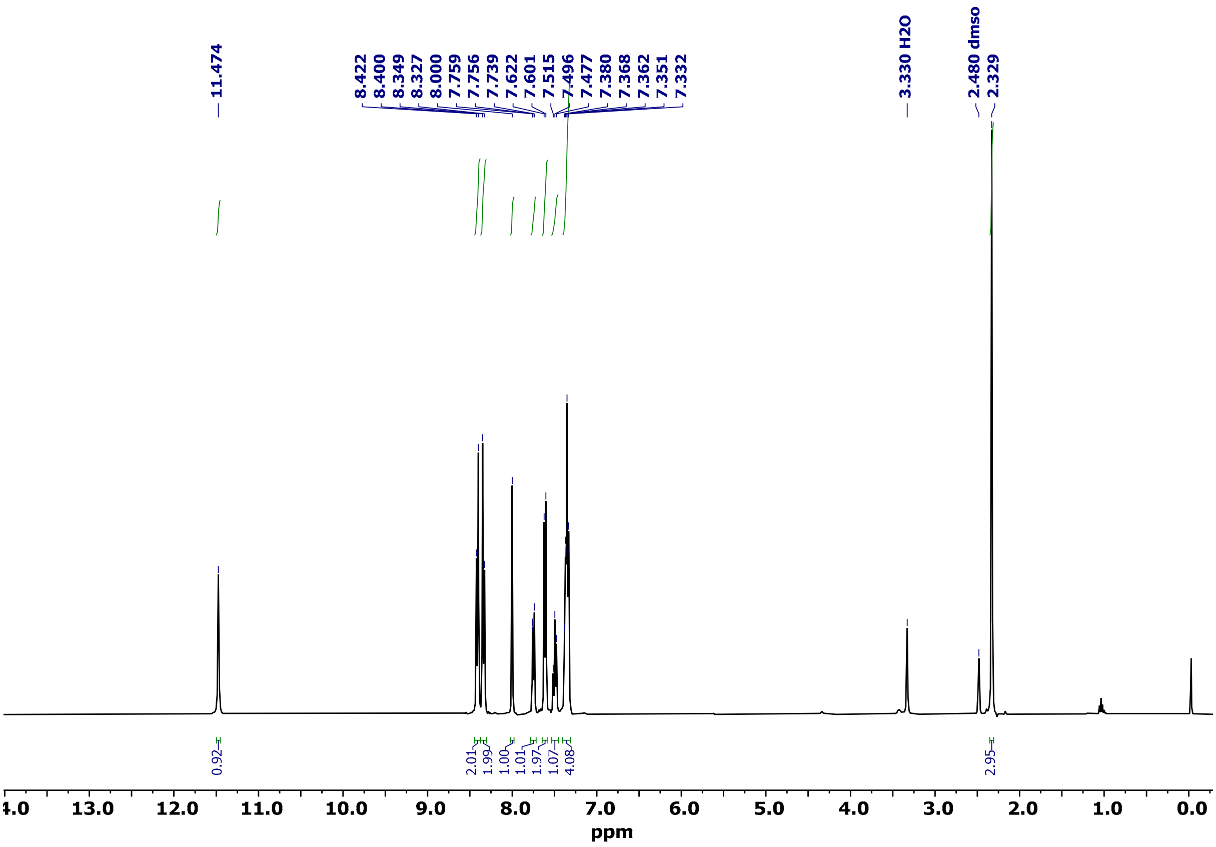

^{13}C NMR spectrum of compound **16**

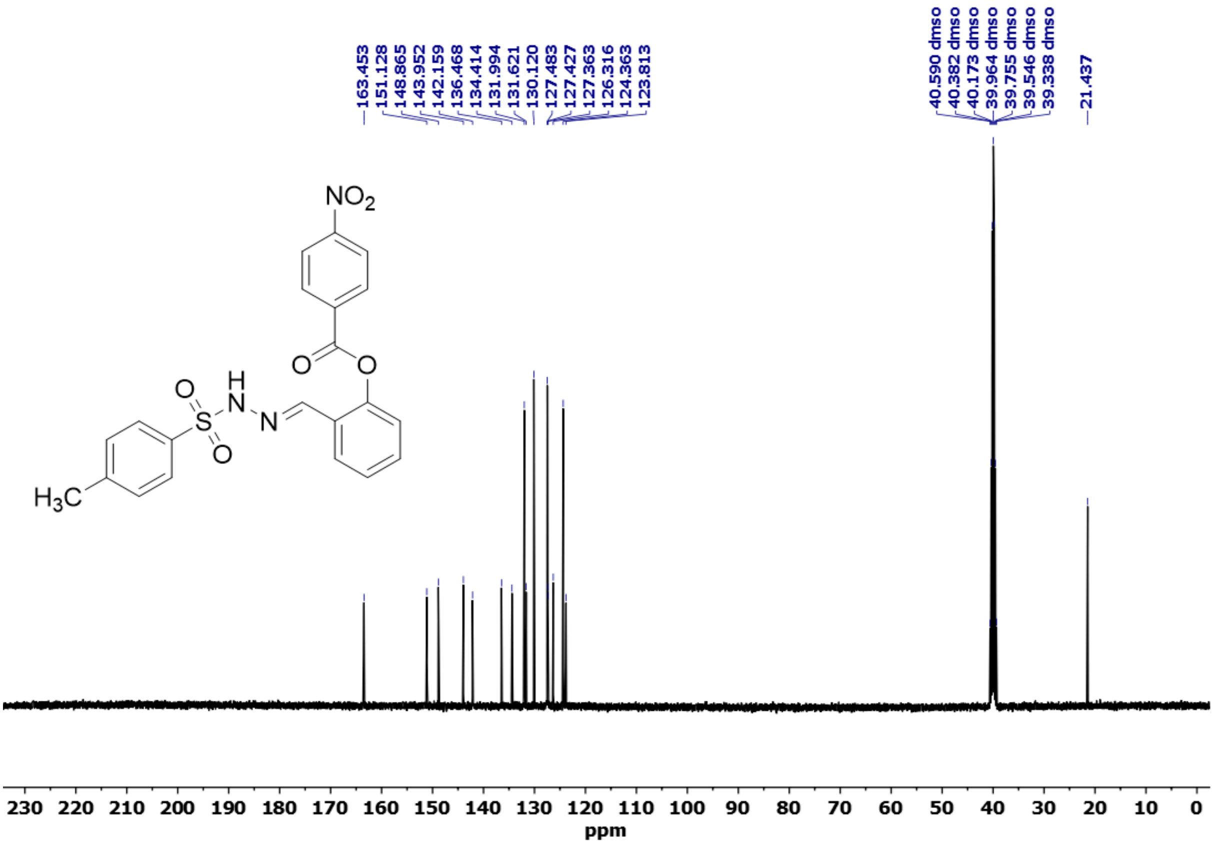

FT-IR spectrum of compound **17**

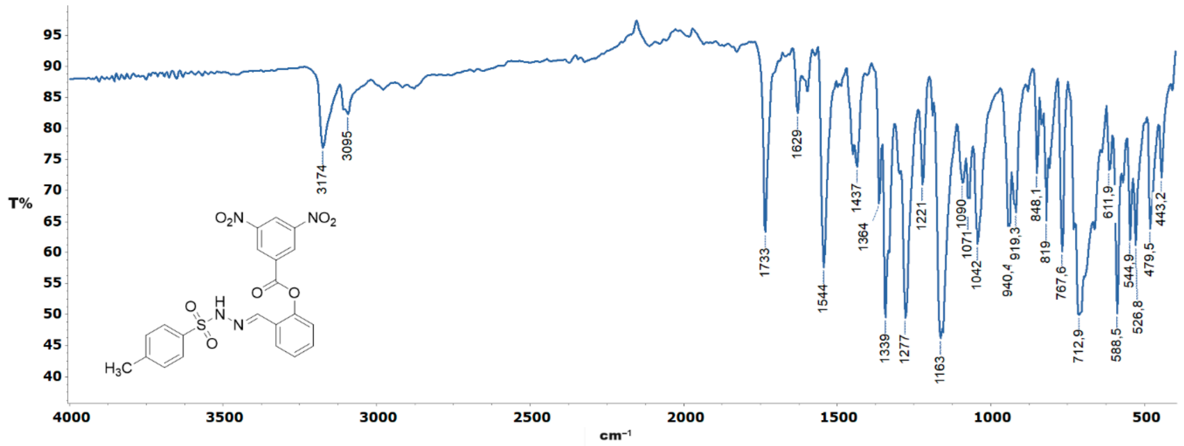

^{1}H NMR spectrum of compound **17**

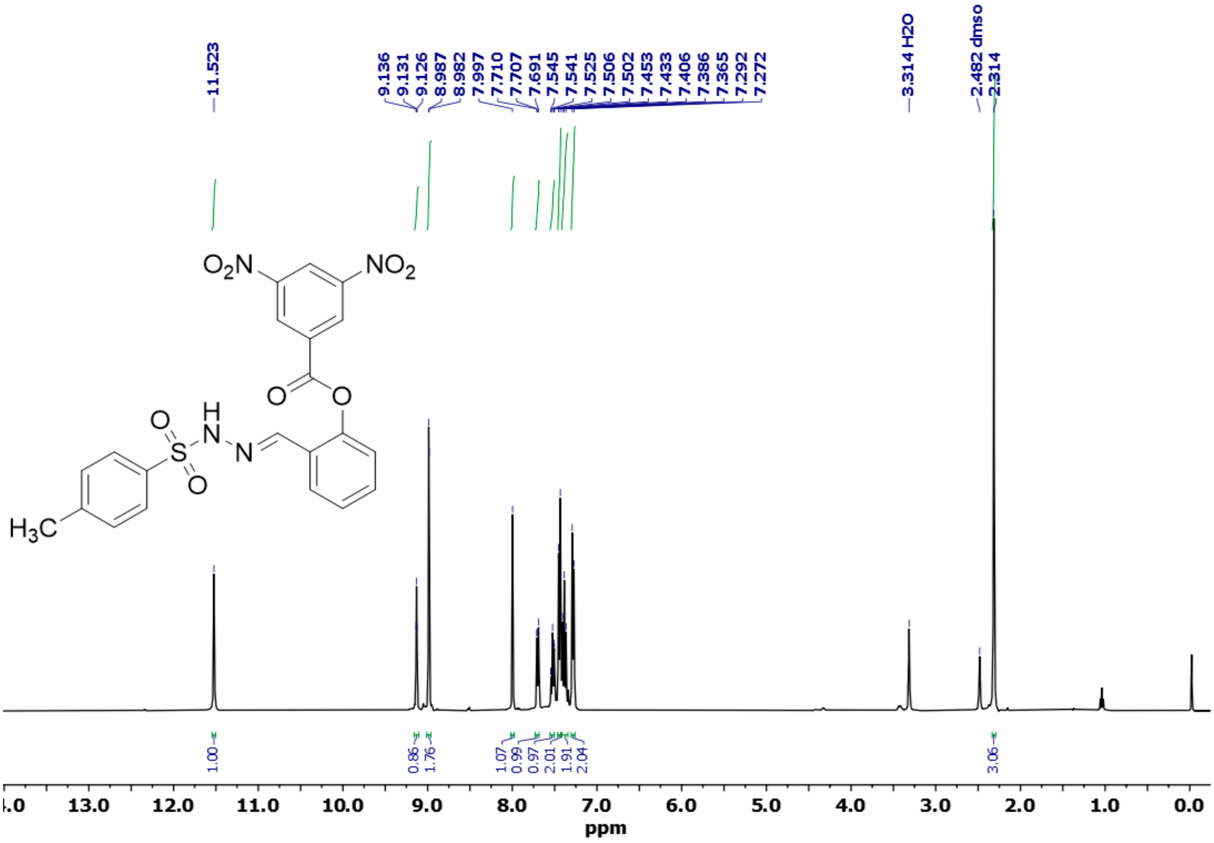

^{13}C NMR spectrum of compound **17**

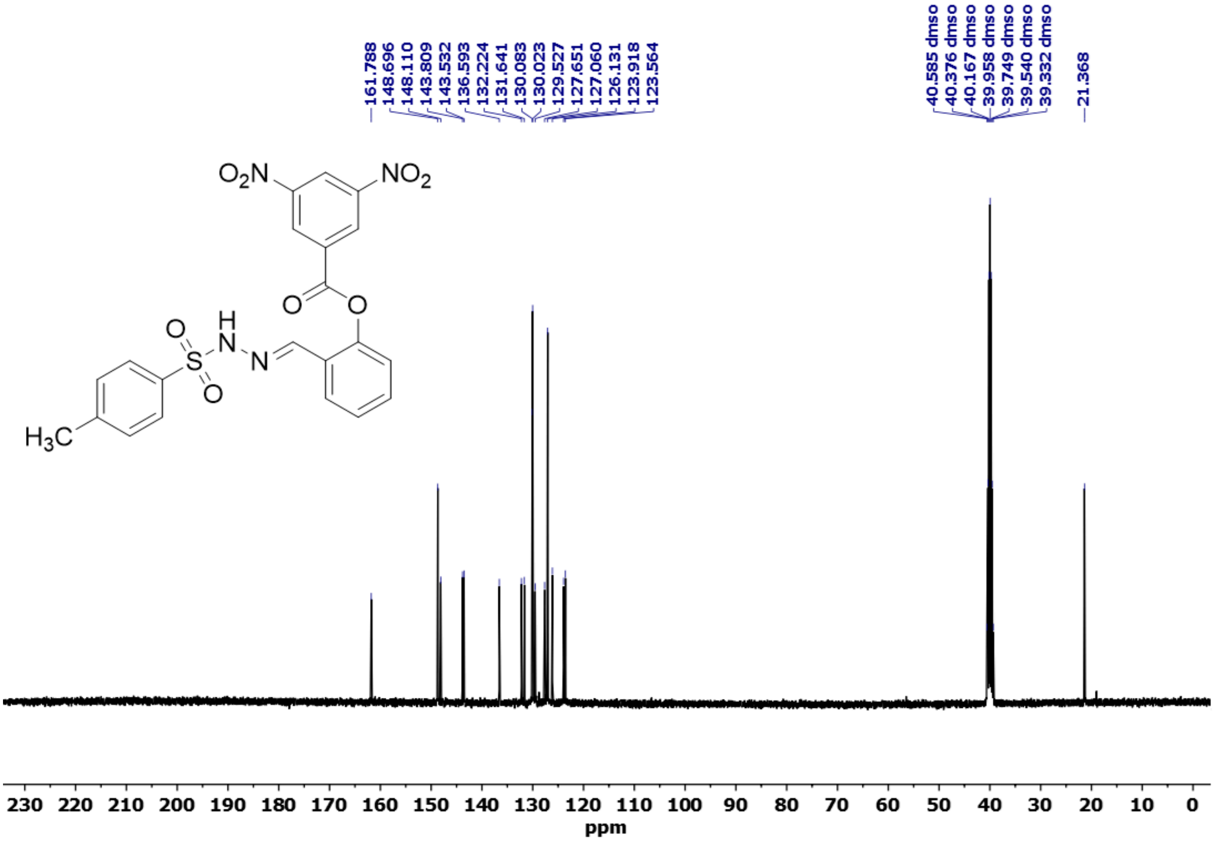

FT-IR spectrum of compound **18**

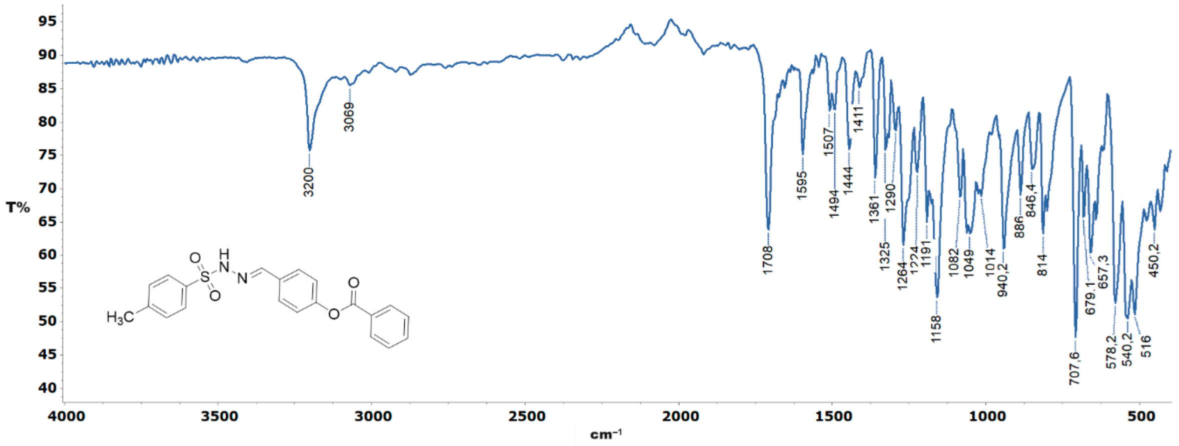

^{1}H NMR spectrum of compound **18**

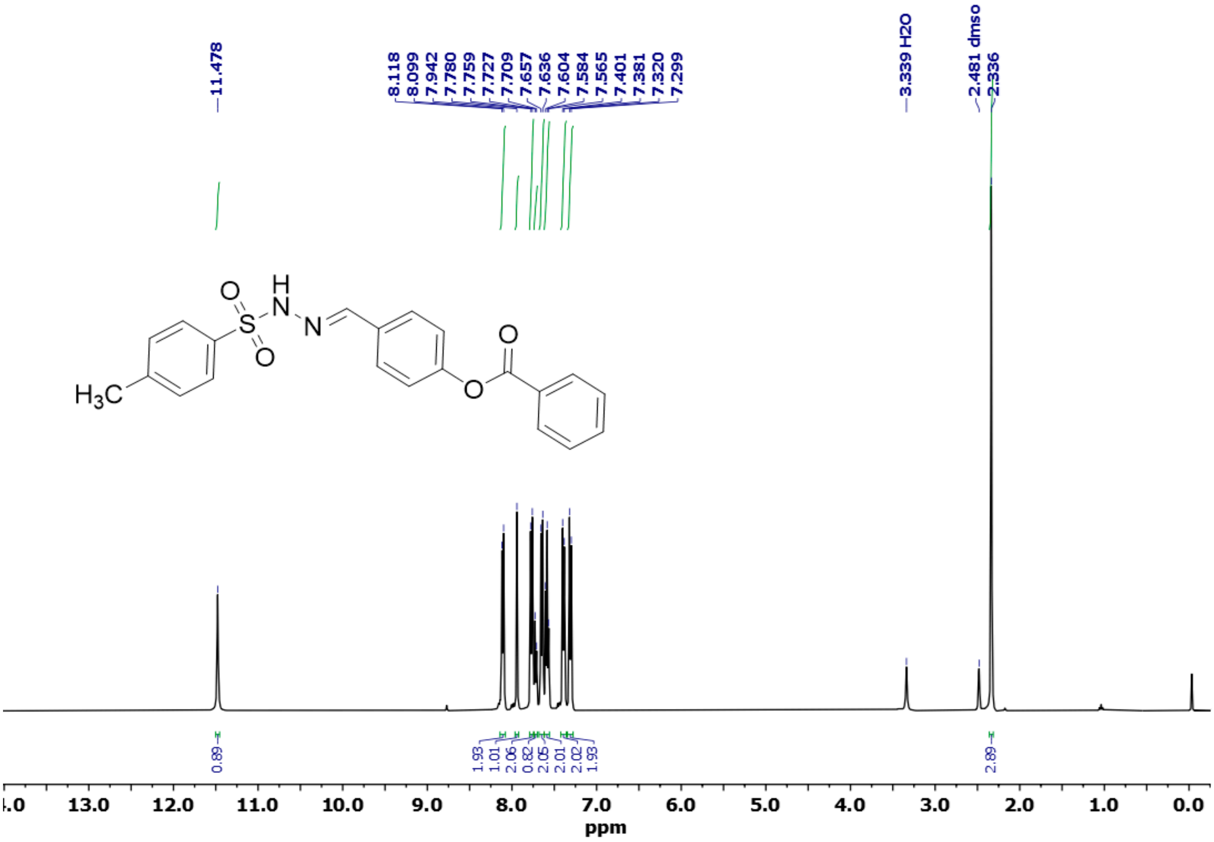

^{13}C NMR spectrum of compound **18**

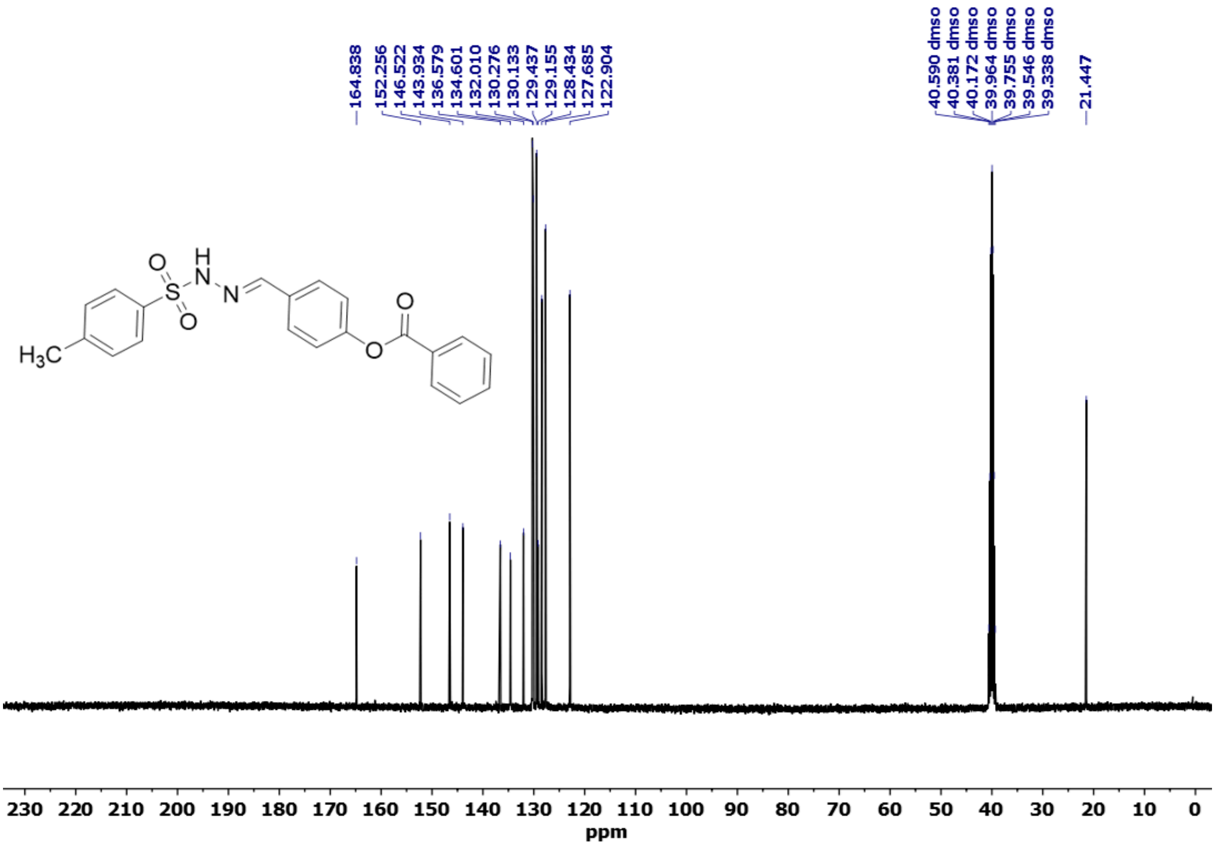

FT-IR spectrum of compound **19**

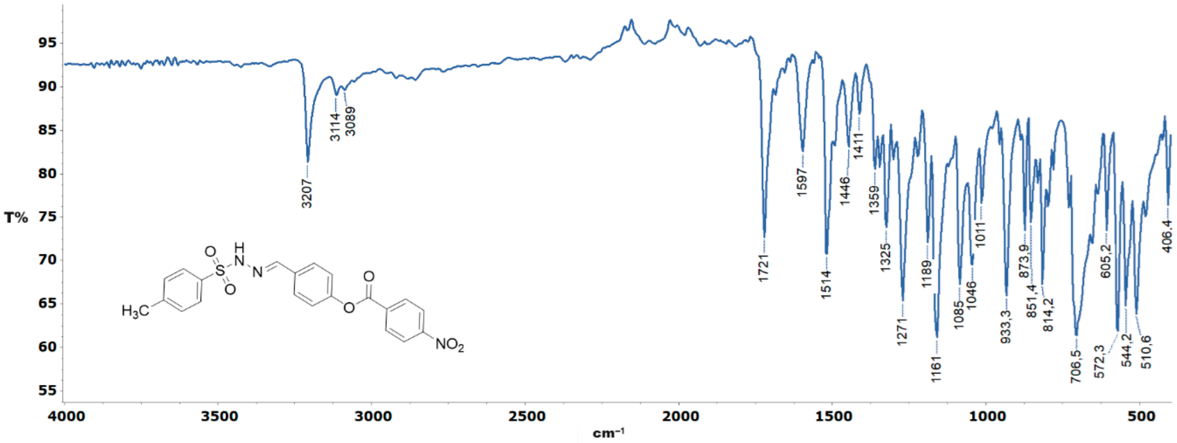

^{1}H NMR spectrum of compound **19**

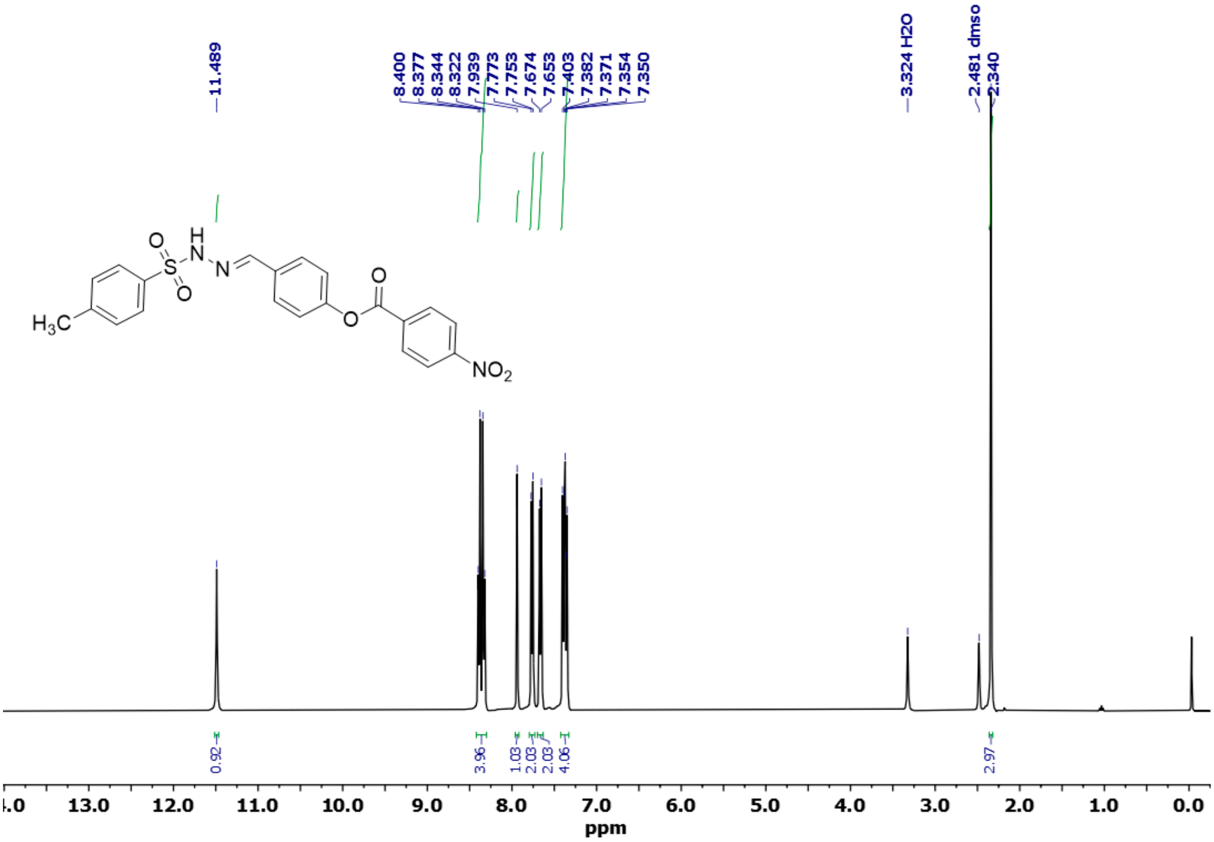

^{13}C NMR spectrum of compound **19**

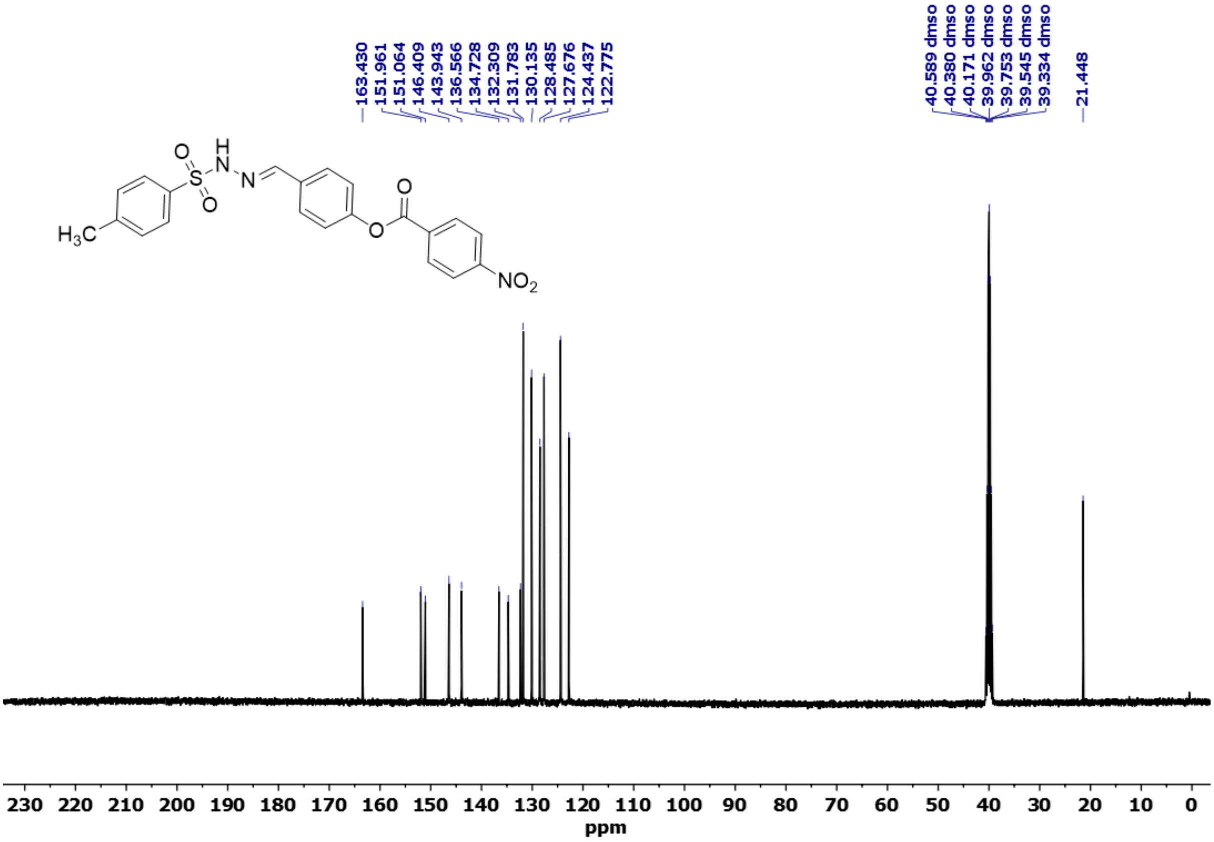

FT-IR spectrum of compound **20**

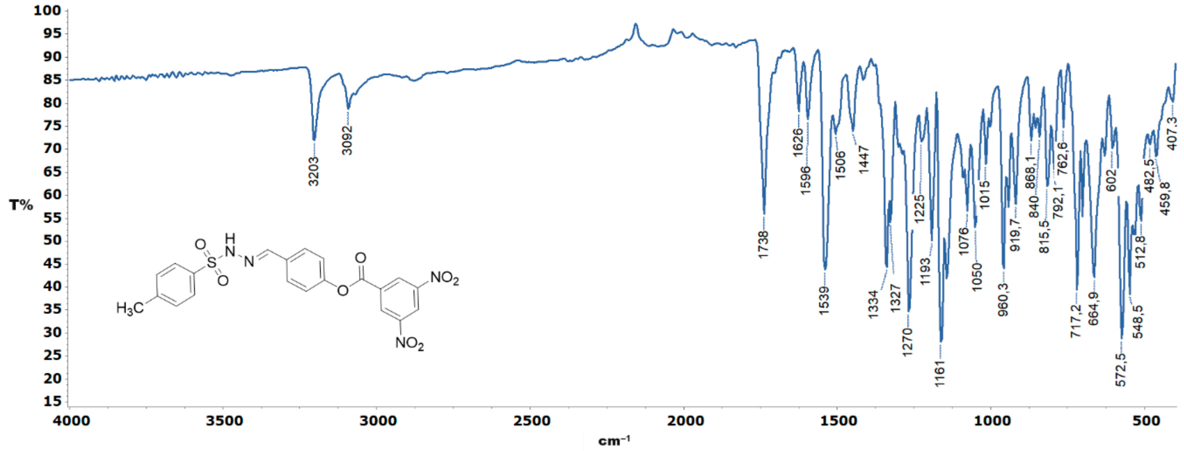

^{1}H NMR spectrum of compound **20**

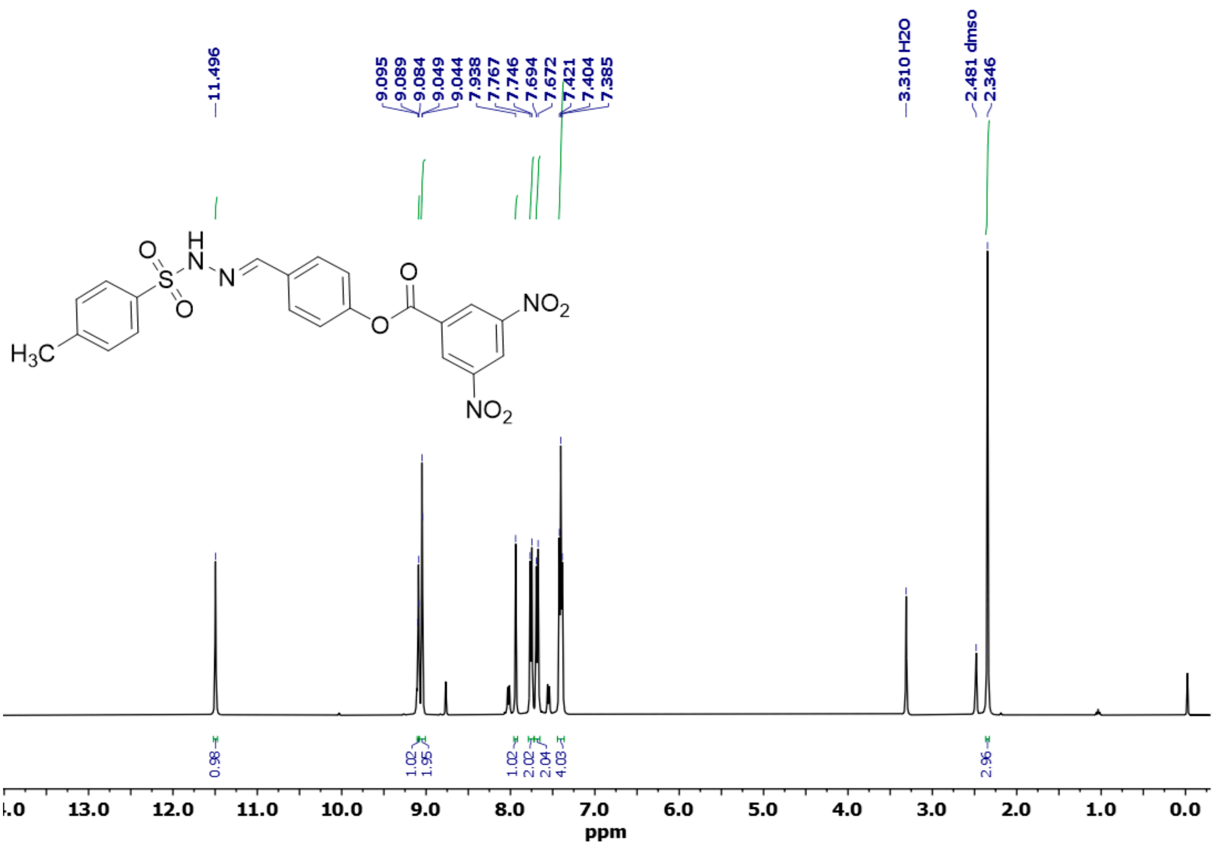

^{13}C NMR spectrum of compound **20**

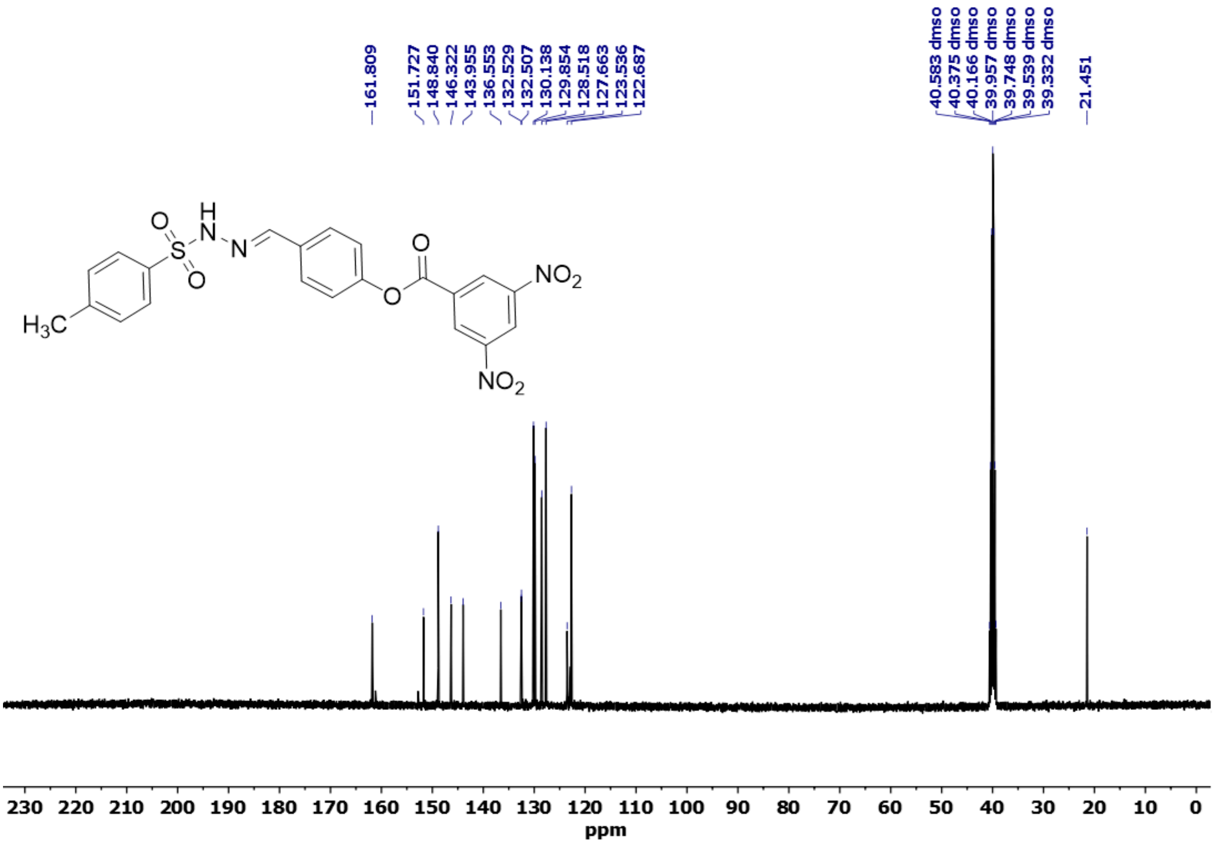

Supplement: Supplementary file 1 [file molecules-29-03478-s001.zip › molecules-3103623-supplementary.pdf]
